# Economic and Environmental Competitiveness of Ethane-Based Technologies for Vinyl Chloride Synthesis

**DOI:** 10.1021/acssuschemeng.3c03006

**Published:** 2023-08-23

**Authors:** Juan D. Medrano-García, Vera Giulimondi, Amedeo Ceruti, Guido Zichittella, Javier Pérez-Ramírez, Gonzalo Guillén-Gosálbez

**Affiliations:** Institute for Chemical and Bioengineering, Department of Chemistry and Applied Biosciences, ETH Zurich, Vladimir-Prelog-Weg 1, 8093 Zurich, Switzerland

**Keywords:** vinyl chloride monomer, ethane chlorination, life cycle assessment, prospective LCA, global warming, process simulation

## Abstract

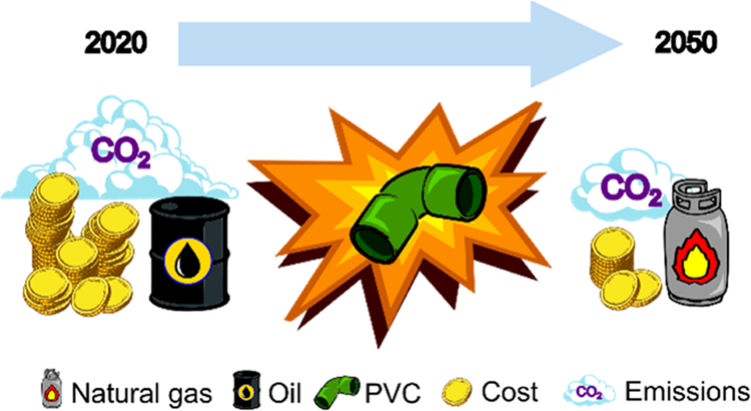

The synthesis of the vinyl chloride monomer (VCM), employed to manufacture poly(vinyl chloride) (PVC) plastic, primarily relies on oil-derived ethylene, resulting in high costs and carbon footprint. Natural gas-derived ethane in VCM synthesis has long been considered a transformative feedstock to lower emissions and expenses. In this work, we evaluate the environmental potential and economics of recently developed catalytic ethane chlorination technologies for VCM synthesis. We consider the ethylene-based business-as-usual (BAU) route and two different ethane-based processes evaluated at their current development level and their full potential, *i.e.*, ideal conversion and selectivity. All routes are assessed under two temporal scenarios: present (2020) and prospective (2050). Combining process simulation and life cycle assessment (LCA), we find that catalytic ethane chlorination technologies can lower the production cost by 32% at their current development state and by 56% when considering their full potential. Though environmentally disadvantageous in the 2020 scenario, they emerge as more sustainable alternatives to the BAU in the 2050 scenario, reducing the carbon footprint of VCM synthesis by up to 26% at their current state and up to 58% at their full potential. Going beyond VCM synthesis, our results highlight prospective LCA as a powerful tool for assessing the true environmental implications of emerging technologies under more decarbonized future energy scenarios.

## Abstract 2

By the year 2050, ethane chlorination technologies are predicted to be the most economically and environmentally advantageous routes for vinyl chloride synthesis.

## Introduction

Poly(vinyl chloride) (PVC) is the third most produced plastic globally, after polyethylene and polypropylene.^1^ Utilizing over 50% of the world’s chlorine production, the global manufacturing of PVC reached 45 Mt in 2018 and is forecasted to grow by *ca.* 2 Mt/year.^2,3^ Meeting such high demand in the future will unavoidably require technologies that can exploit both carbon and chlorine sources sustainably. PVC results from the radical-based polymerization of the vinyl chloride monomer (C_2_H_3_Cl, VCM). Currently, two processes are employed on a commercial scale for VCM synthesis. The main technology, accounting for two-thirds of the global PVC production,^1,3,4^ relies on oil-derived ethylene (C_2_H_4_), entailing a high carbon footprint of 2.00 kg of CO_2_ equivalent per kg of VCM (2.00 kg(CO_2_-eq) kg(VCM)^−1^).^5^ Furthermore, the ever-increasing oil price is driving this process toward economic unfeasibility, especially in the Western world.^6^ The second commercial process for VCM synthesis is the hydrochlorination of coal-derived acetylene (C_2_H_2_). However, this technology relies on toxic and volatile mercuric chloride catalysts, emitting 40 tons of mercury per year and posing serious threats to the environment and population.^1,3^

In the quest to identify more sustainable feedstocks, ethane (C_2_H_6_) has long emerged as the primary candidate because of its reduced carbon footprint (1.01 kg(CO_2_-eq) kg(ethane)^−1^*vs*. 1.52 kg(CO_2_-eq) kg(ethylene)^−1^).^5,7−10^ Despite extensive research efforts, no catalytic process has been implemented on an industrial scale, owing to intrinsic challenges stemming from the kinetically hindered activation of alkanes and reaction intermediates.^11,12^ In 2010, Dow piloted a catalytic 1-step (oxy)chlorination process converting ethane in the presence of hydrogen chloride (HCl), oxygen (O_2_), and chlorine (Cl_2_) into VCM over a lanthanum-based catalyst.^13,14^ It exhibited up to 80% VCM selectivity and productivity of 0.05 kg(VCM) h^–1^ kg(cat)^−1^. Still, the formation of water (H_2_O) and carbon oxides (*i.e.*, CO_*x*_, *x* = 1, 2) is inevitable when utilizing O_2_, inherently decreasing the efficiency of the process as these byproducts cannot be recycled.

To overcome these hurdles, we recently demonstrated a promising strategy based on reacting ethane with Cl_2_ at mild temperatures (≥250 °C) and atmospheric pressure in the absence of O_2_.^15^ Specifically, we reported a chlorination process selectively converting ethane into 1,2-dichloroethane (C_2_H_4_Cl_2_, EDC, up to 90%) over a europium-based catalyst. This route can produce VCM upon thermal cracking of EDC, attaining order-of-magnitude higher productivity (0.4 kg(VCM) h^–1^ kg(cat)^−1^) than state-of-the-art oxychlorination technologies. This approach is particularly attractive for Western countries that have large natural gas resources and chlorine production.^16−18^ Exploratory economic and environmental analyses were recently conducted to compare the ethane chlorination technology with the commercial ethylene-based process, considering the full potential of both routes, *i.e.*, full conversion and selectivity without separation.^19^ Reductions in production cost (45%) and carbon footprint (20%) were predicted, favoring the ethane-based route.

In the wake of these promising results, we herein conduct in-depth comparative economic and life cycle assessment (LCA) analyses of the ethane chlorination and business-as-usual (BAU) processes to explore the full scope of the former and accurately assess its prospects. To this end, we perform process simulations considering the BAU and two ethane-based technologies, which consist of a 2-step process and a 1-step process, directly yielding VCM. The latter ones are evaluated under different technological scenarios: considering the current development level and the potential future state attaining full conversion and product selectivity to VCM. Finally, we assess the economics and sustainability of the BAU and the ethane-based technologies under current and 2050 prospective scenarios (*i.e.*, by considering future decarbonization trends in the background data employed in the LCA calculations, which are often assumed constant). Our results demonstrate that while ethane-based technologies still rely on fossil carbon, they could help to cope with the ever-increasing PVC demand and depleting oil resources while reducing carbon emissions in the transition toward fully sustainable plastics manufacture.

## Methodology

Five different routes for VCM synthesis were considered and modeled in Aspen Plus v12. These simulations represent the BAU ethylene chlorination balanced process, the scaling (*i.e.*, ex ante analysis) of the 1-step and 2-step ethane-based routes from experimental laboratory data (real scenario), and the potential of said routes assuming 100% conversion and selectivity (ideal scenario). Details on the catalyst preparation and catalytic tests are provided in the Supporting Information (Section A). The laboratory-to-industrial scale-up was carried out following standard practices.^20,21^ From these simulations and after a heat integration analysis performed in Aspen Energy Analyzer, we obtain the material and energy inputs and outputs associated with VCM synthesis (*i.e.*, data in the foreground system). With these data, we then proceed to carry out an LCA of the different scenarios in Brightway2 v.2.4.2, taking the background data from Premise v1.3.2 databases based on Ecoinvent v3.8.^5,22,23^ Finally, we perform an economic assessment using the variable operating costs (raw materials and utilities) of the plant. We present first the various scenarios herein examined, followed by a description of the process simulations, the LCA, and the economic analysis.

### Case Studies

**Figure 1:**
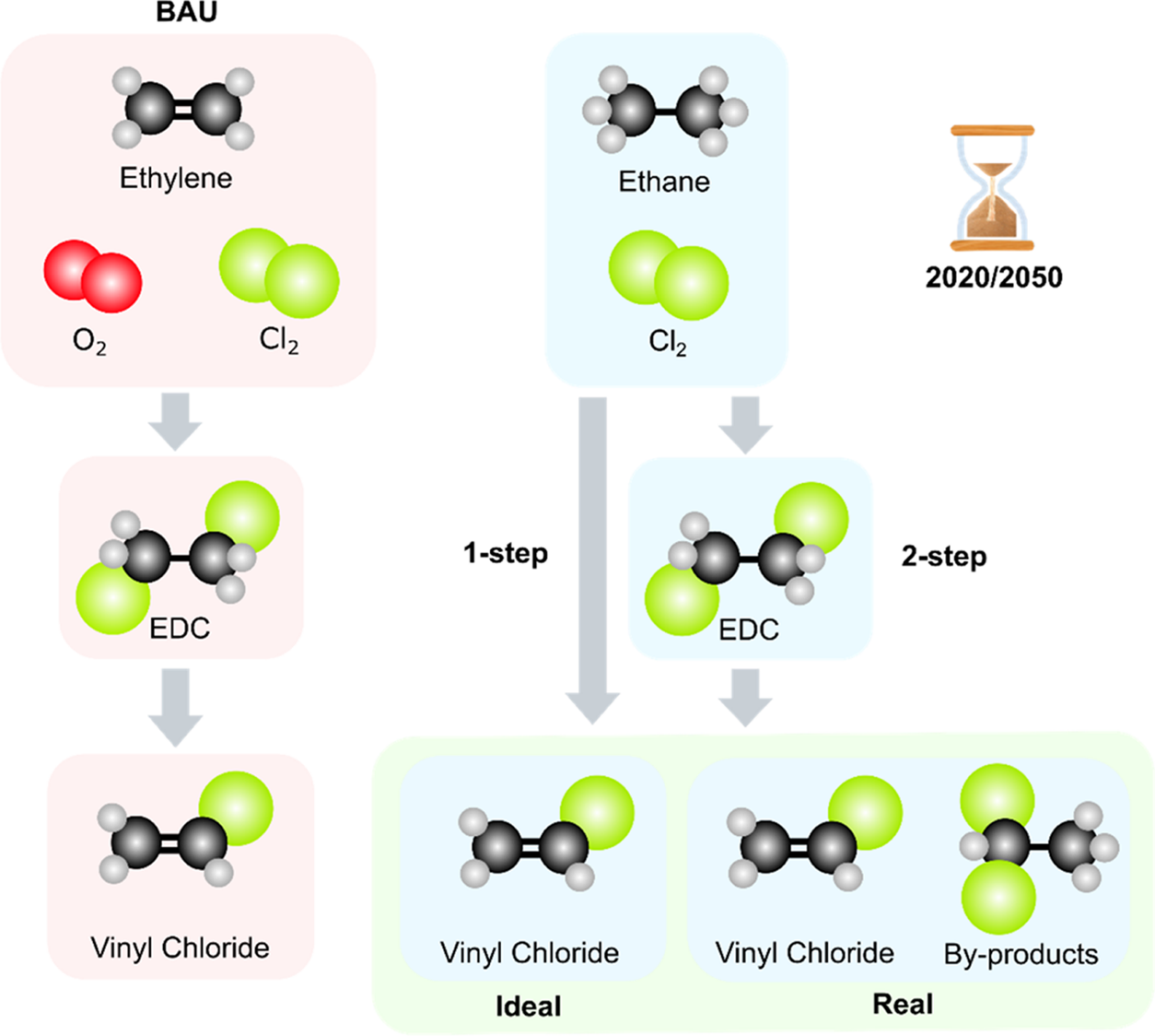
Overview of the process (BAU, 2-step and 1-step ethane chlorination), technological (ideal with 100% conversion and selectivity; real with experimental catalytic results), and temporal (2020 and 2050) scenarios evaluated in this work.

We consider 10 scenarios (Figure 1) consisting of the combinations of the three VCM synthesis technologies (BAU, 1-step, and 2-step) and the two ideal ethane-based processes (1-step ideal and 2-step ideal) under two temporal scenarios that evaluate the environmental performance in the present (2020 scenario) and the future (2050 scenario). The databases for the scenarios were created using projections from the Integrated Assessment Model (IAM) IMAGE.^24^ More specifically, they were built considering the Paris Agreement scenario of limiting global warming to a 1.5 °C increase in comparison to preindustrial levels by 2100.

### VCM Synthesis Simulation Overview

**Figure 2:**
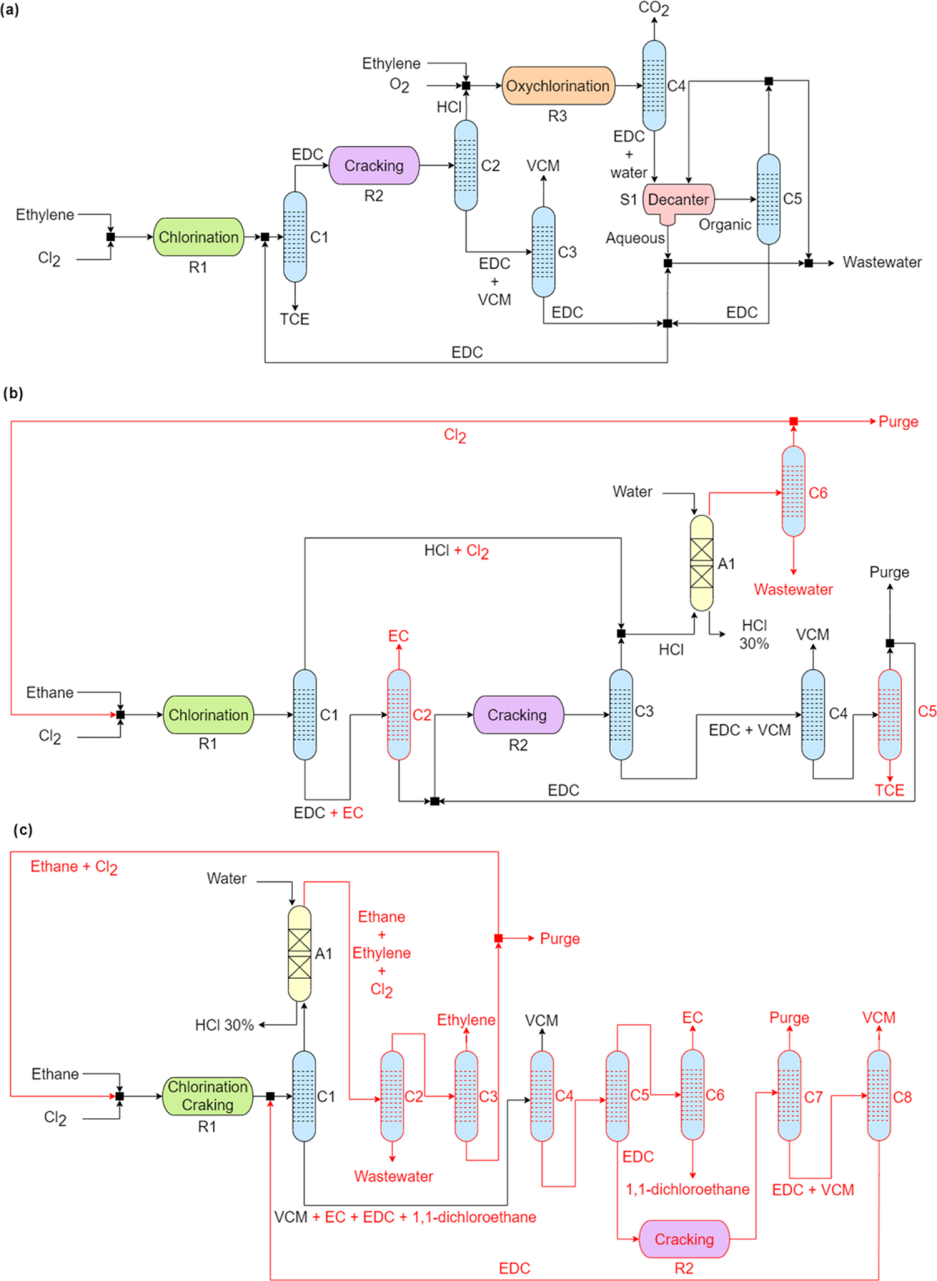
Simplified process flowsheets for VCM synthesis: (a) ethylene route (BAU); (b) 2-step ethane route; and (c) 1-step ethane route. For panels (b, c), darker sections indicate the ideal process configurations (units in red would be omitted in those cases).

In this section, we describe the five process simulations used in the sustainability assessment of VCM synthesis. The simplified process flowsheets are shown in Figure 2. More details on the process conditions, chemical reactions, and results of the simulations are provided in the Supporting Information (Sections B and C).

The ethylene BAU route (Figure 2a) is simulated considering the current state-of-the-art technology.^25,26^ Here, ethylene and Cl_2_ enter the chlorination reactor (R1) to produce EDC. The resulting trichloroethane (C_2_H_3_Cl_3_, TCE) byproduct is removed in the first column (C1), while the rest of the products enter the cracking reactor (R2), where HCl and VCM are obtained. In the second column (C2), HCl is removed from the VCM and unreacted EDC. The liquid stream is then sent to the third column (C3), where VCM (300 kt/y) is retrieved as the distillate product and EDC is recycled back to C1. On the other hand, HCl removed in C2 is mixed with more ethylene and O_2_. The stream enters the oxychlorination reactor (C3), where EDC is produced. Undesired CO_2_ is removed in the fourth column (C4), while the bottom product is sent to a decanter (S1). Here, the organic phase is sent to the fifth column (C5), where dry EDC is obtained as the bottom product and recycled back to C1. The distillate is partially purged and sent back to S1. The aqueous phase of the decanter (S1) is retrieved as wastewater.

The 2-step ethane route (Figure 2b) is modeled based on the experimental data reported by Zichittella et al.^15^ Ethane and Cl_2_ produce EDC in the chlorination reactor (R1) along with the ethyl chloride (C_2_H_5_Cl, EC) and TCE byproducts. In the first column (C1), HCl and unreacted Cl_2_ are obtained as the top product and sent to the adiabatic absorber (A1), where water at 25 °C is used to wash the stream and HCl is recovered as a 30 wt % solution in water at 80–90 °C. The wet Cl_2_ steam is dried by water condensation (C6), and the gas is recycled back to the entrance of R1. The bottom product of C1 is sent to a second column (C2), where EC is separated as a distillate, and the bottom stream, mainly composed of EDC, is sent to the cracking reactor (R2). Here, VCM and HCl are produced. After the third distillation column (C3), HCl is sent to A1, while the bottom product enters the fourth distillation column (C4), where VCM is retrieved as the top product and a mixture of EDC and TCE is sent to the fifth and final column (C5), where TCE is obtained as the bottom product, and the distillate, mainly containing EDC, is recycled back to R2 after a small purge.

The ideal version of the 2-step ethane route follows the same steps as its real counterpart; however, C2, C5, and C6 are omitted owing to the chlorination reactor being 100% selective toward EDC production. The goal here is to evaluate the full potential of such a route, assuming an ideal catalyst.

The 1-step ethane route simulation (Figure 2c) is based on experimental data, as detailed in the Supporting Information (Section A). Ethane and Cl_2_ enter the chlorination/cracking reactor (R1), where VCM, EDC, EC, 1,1-dichloroethane, HCl, and ethylene are formed. The reaction products are sent to the first distillation column (C1), where HCl, Cl_2_, ethane, and ethylene are retrieved as the top product. The stream is sent to an absorber (A1), where HCl is removed with water (30 wt %). The rest of the gases are dried in the second column (C2), and ethylene is separated as a distillate in the third distillation column (C3), working under cryogenic conditions. After a purge, the ethane and Cl_2_ bottom product is recycled back to R1. The bottom product of C1 is fed into the fourth column (C4), mainly containing VCM, EDC, EC, and 1,1-dichloroethane. Here, VCM is obtained as the top product. The rest of the components enter the fifth column (C5), where EDC is separated in the bottom and sent to a cracking reactor (R2) to produce additional VCM and HCl. The distillate is then sent to the sixth column (C6), where EC and 1,1-dichloroethane are retrieved as the top and bottom products, respectively. The effluent of R2 is sent to the seventh column (C7) to remove impurities. The bottom stream of C7 finally enters the eighth column (C8), where more VCM is obtained as the distillate and EDC is recovered as the bottom product, and is then sent back to C1.

The ideal simulation of the 1-step ethane route considers full conversion and selectivity toward the VCM in the chlorination/cracking reactor. For this reason, the process only requires the first distillation column to separate VCM and HCl.

### Environmental Assessment

The LCA is carried out following the phases described in the ISO 14040/44 framework.^27^ The first phase consists of the goal and scope definition, where we consider a cradle-to-gate assessment of the synthesis of VCM. We include all upstream activities, which were obtained from Ecoinvent v3.8 and Premise v1.3.2.^5,22^ As mentioned in a previous section, the future scenario is based on the Paris Agreement limited temperature increase of 1.5 °C by 2100, and it is built using the IMAGE IAMS. Under this assumption, the databases are updated with new power generation, cement and steel production, and transport inventories, also including the expected evolution of process efficiencies (*e.g.*, improved photovoltaic panels for electricity generation), shares (*e.g.*, increased contributions of renewables with the years), and markets (*e.g.*, residual or purpose-grown biomass for power generation). The chosen functional unit is 1 kg of VCM.

During phase two, we build the life cycle inventories (LCIs). Our foreground system is based on the material and energy stream results of the Aspen Plus simulations, while the background system is defined from the Ecoinvent and Premise databases accessed through Brightway2 v2.4.2.^23^ Finally, in the third phase, we use the ReCiPe v1.1 methodology to calculate the life cycle impact assessment (LCIA) from the LCIs again using Brightway2 v2.4.2.^28^

### Economic Assessment

The production cost per kg of VCM is calculated based on the raw material and utility consumption of the plant, assuming that the effect of the capital cost is negligible due to economies of scale (Section D of the Supporting Information) and the high operating costs eqs 1 and 2, as it was found in other petrochemical processes^29−31^
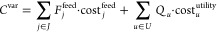
1
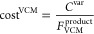
2where *C*^var^ is the variable operating cost of the plant [$ h^–1^]; *J* is the set of raw materials (*i.e.*, mass inputs) involved in the synthesis of VCM; *F*_*j*_^feed^ is the mass flow [kg h^–1^] of raw material *j*; *U* is the set of utilities required for the synthesis of VCM, including heating, cooling at 20 to 25 °C, refrigeration at −50 °C, refrigeration at −125 °C, and electricity; *Q*_*u*_ is the energy flow [MW] of utility *u*; cost_*j*_^feed^ and cost_*u*_^utility^ are the prices of raw material *j* [$ h^–1^] and utility *u* [$ MW^–1^], respectively; *F*_VCM_^product^ is the mass flow [kg h^–1^] of the product; and cost^vcm^ is the production cost of VCM [$ kg^–1^].

The majority of global ethane production relies on natural gas processing and fractionation;^32^ hence, their economics are also closely related. Given the volatility of natural gas prices due to recent events such as the global pandemic or geopolitical conflicts, it is expected that ethane would also be affected. For this reason, the potential variability of the ethane price and its impact on the VCM production cost are investigated.

## Results and Discussion

The simulation results, in the form of inlet and outlet materials and energy flows per kg of VCM produced, and the data employed in the economic study are reported in the Supporting Information (Sections C and D, respectively). Here, we analyze the environmental and economic results.

### Environmental Results

**Figure 3:**
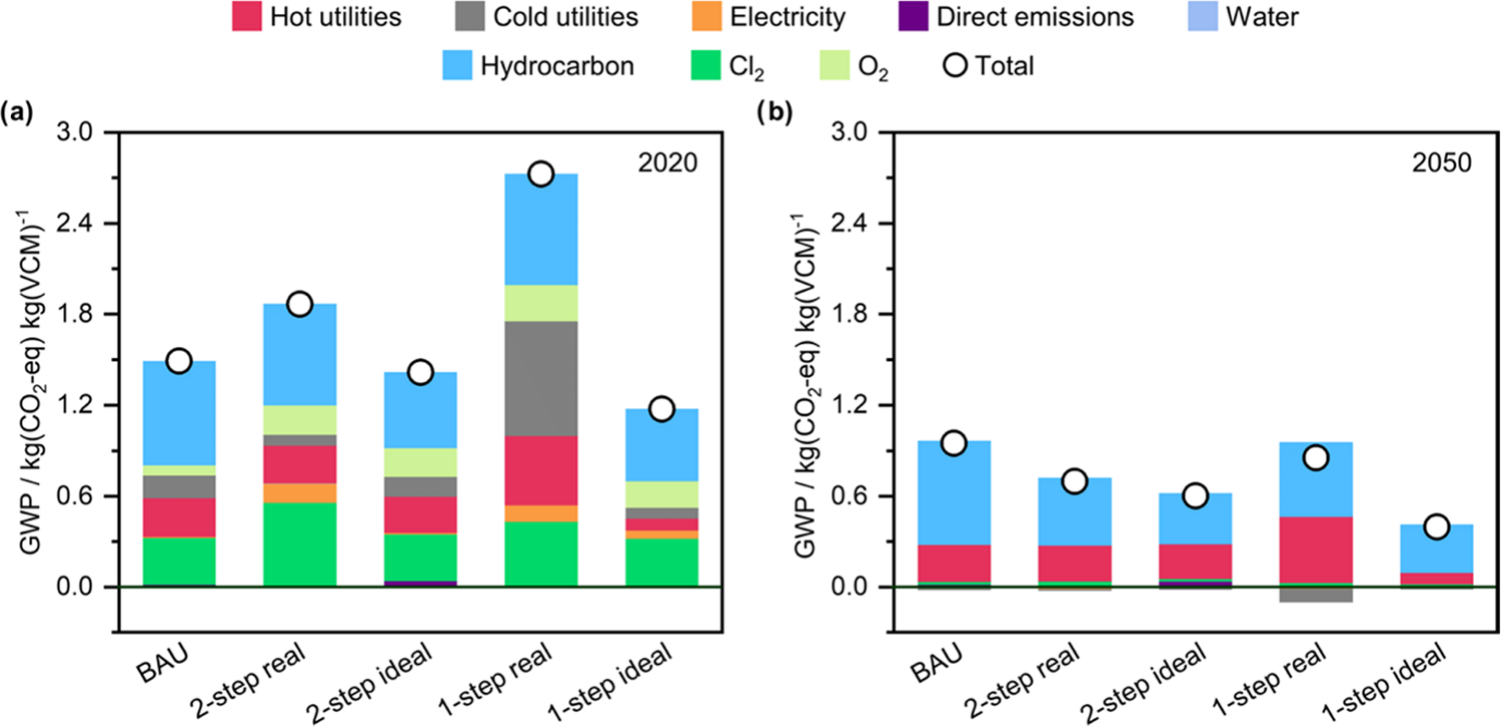
Climate change impact breakdown of the five routes for VCM synthesis (BAU, 2-step, and 1-step, considering both the real and ideal scenarios) for both (a) present and (b) future temporal scenarios. The “hydrocarbon” contribution refers to ethylene and ethane for the BAU and ethane-based route scenarios, respectively.

As shown in Figure 3, although the BAU exhibits a lower global warming impact than both emerging technologies at the current development level and energetic scenario, the superior sustainability of the ethane-based routes compared with the BAU is evident when considering the prospective 2050 scenario. Furthermore, the performance (ideal scenario) of the emerging technologies also surpasses that of the BAU under the current energetic 2020 scenario. As discussed next, these disparities are due to the expected decarbonization of the energy inputs, whose carbon footprint will be dictated by the future power mix, designed bearing in mind the climate goals.^33^

In the 2020 scenario, the BAU shows an impact of 1.87 kg(CO_2_-eq) kg(VCM)^−1^, mainly led by the raw materials, ethylene (46%), Cl_2_ (20%), and O_2_ (4%), followed by the utilities, heating (17%), and cooling (10%). The 2-step ethane-based route, with an impact of 1.49 kg_CO_2_-eq_ kg(VCM)^−1^ (25% increase in comparison with the BAU), presents a similar behavior, with raw materials making up the largest share (ethane, Cl_2_, and O_2_ with 36, 30, and 10%, respectively), followed by the utilities (heating, electricity and cooling with 13, 7, and 4%, respectively). In contrast, the main contribution to the total climate change impact in the 1-step route (2.73 kg(CO_2_-eq) kg(VCM)^−1^, 83% increase) is cooling (28%), followed by ethane (27%), heating (17%), Cl_2_ (16%), O_2_ (9%), and general process electricity for compression and pumping (4%). The high amounts of cooling in the 1-step route are a consequence of ethylene being produced as a byproduct and its subsequent separation from unreacted ethane and Cl_2_, which requires cryogenic distillation below −100 °C. The high electricity demand to operate the cryogenic cycle (0.83 kWh MW^–1^ cooling)^34,35^ appreciably impacts the overall environmental performance of the route due to the heavy-fossil-fuel-reliant current electricity mix (0.33 kg(CO_2_-eq) kW^–1^).

However, in the future scenario, the electricity mix is much more decarbonized due to the deployment of bioenergy with carbon capture and storage (BECCS), leading to a carbon-negative power supply (−0.04 kg(CO_2_-eq) kW^–1^).^36^ Recall that this scenario is based on the 2050 projection of the IMAGE IAM, which assumes that global warming in 2100 will not surpass 1.5 °C in comparison with preindustrial levels. Such a scenario leads to an impact of 0.85 and 0.70 kg(CO_2_-eq) kg(VCM)^−1^ for the 1- and 2-step ethane routes, respectively, compared with 0.95 kg(CO_2_-eq) kg(VCM)^−1^ for the 2050 BAU. Therefore, the ethane-based process could outperform the BAU without further optimizing the technology. In this scenario, the carbon source is the main contributor to the impact, with ethylene being 73% of the impact in the BAU and ethane totaling 57 and 64% for the 1- and 2-step routes, respectively, followed by heating, with 26, 51, and 34% for the three synthesis routes. The remaining contributions are minor, with Cl_2_ only accounting for 2, 3, and 4% for the BAU and 1-step and 2-step processes, respectively. Specifically, this drastic reduction is tied to the contribution from Cl_2_, whose footprint is mostly dictated by the power input to the electrolysis in the chloralkali process. Finally, it is worth noting that cooling provides a negative net impact contribution due to the carbon-negative electricity mix. Regarding heating, its large contribution is given by the assumption that both today and in 2050, the heating requirements would be covered using fossil fuels (*e.g.*, natural gas).

The ideal 1- and 2-step ethane routes outperform all other process configurations regardless of the temporal scenario assessed. In the 2020 scenario, the ideal 2-step route has an associated impact of 1.42 kg(CO_2_-eq) kg(VCM)^−1^, while the ideal 1-step route is 1.18 kg(CO_2_-eq) kg(VCM)^−1^ (5 and 43% decrease compared to the BAU, respectively). Similarly to the real routes, most of these impacts stem from the raw materials: 35–40% from ethane, 22–27% from Cl_2_, and 13–15% from O_2_, while the rest is mainly attributed to heating (7–17%), cooling (6–9%), and electricity (1–4%). In the 2050 scenario, the impact gap between the ideal and real scenarios grows, with reductions of 58% for the 1-step route (0.40 kg(CO_2_-eq) kg(VCM)^−1^) and 36% for the 2-step (0.60 kg(CO_2_-eq) kg(VCM)^−1^). Here, ethane is the main contributor to the global warming impact, with 72 and 55% for the 2-step and 1-step routes. In the BAU (0.95 kg(CO_2_-eq) kg(VCM)^−1^), ethylene is responsible for 73% of the total impact. Overall, these results highlight the environmental benefit of developing technologies enabling the use of ethane instead of ethylene as feedstock for VCM synthesis, halving the carbon footprint (0.67 kg(CO_2_-eq) kg(VCM)^−1^*vs.* 1.52 kg(CO_2_-eq) kg(VCM)^−1^, respectively, in the 2050 scenario). Raw materials and heating are responsible for over 90% of the VCM synthesis impacts since, in all of the results, they are linked to fossil resources, even in the future scenario. Hence, moving toward a renewable source of ethane and electrifying industrial heating will be key to further decarbonizing the monomer synthesis in the future.

Catalyst design strategies dispersing the active metal phase on suitable carriers or modifying the active phase with metal promoters hold promise to maximize performance.^4,15^ Still, attaining full product yield might not be necessary to achieve environmental competitiveness with the BAU. Thus, we calculated the product selectivity required for both the 2- and 1-step processes to environmentally outperform the BAU. Specifically, we evaluated improvements in the product selectivity while ethane conversion is maintained constant. The total emissions (kg CO_2_-eq) are a function of the selectivity since most heating and cooling (refrigeration) are associated with byproduct separation. Therefore, as the selectivity increases, the burdens associated with these utilities decrease. Accordingly, we estimate that the selectivities of EDC and VCM required for the 2- and 1-step ethane routes to be environmentally competitive with the BAU are 89 and 85%, respectively. Finally, reactor design can also play a key role in improving the overall performance. For instance, future modeling studies are encouraged to explore the effect of Cl_2_ stage feeding, aiming to keep a high ethane conversion while reducing side reactions. Combining both catalyst and reactor design strategies offer a promising approach to close the remaining 14 and 22% selectivity gap, respectively, for the 2- and 1-step processes.

### Economic Results

**Figure 4:**
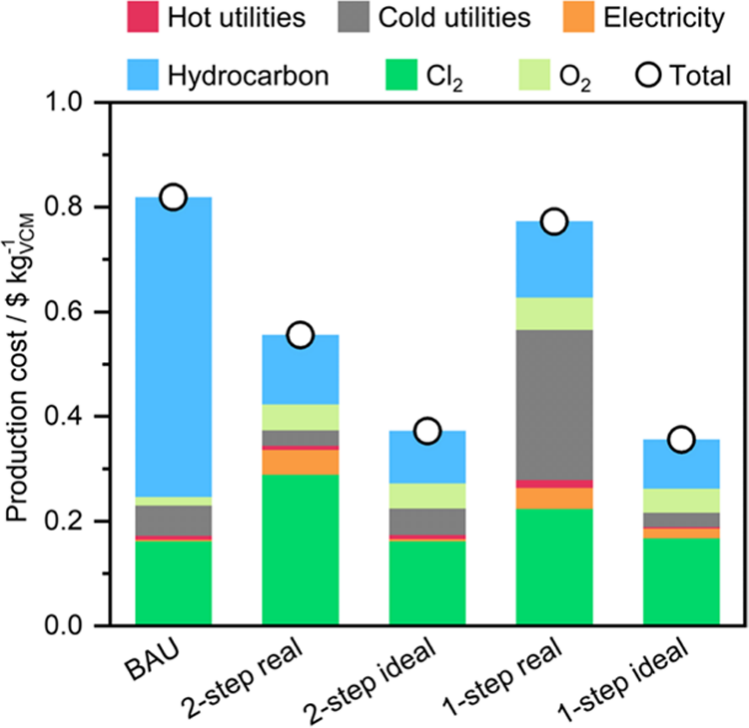
Economic results of the five routes for VCM synthesis (BAU, 2-step, and 1-step, considering both the real and ideal scenarios). The economic parameters used are based on data from 2019.^37^ The “hydrocarbon” contribution refers to ethylene and ethane for the BAU and ethane-based route scenarios, respectively.

Figure 4 shows the economic results of the ethane-based VCM synthesis routes (1-step and 2-step real and ideal) compared with the BAU.

At first glance, all ethane-based routes are currently competitive with the BAU, independently of the level of readiness assessed. This outcome is mainly attributed to the price disparity between ethylene (1.26 $ kg^–1^) and ethane (0.20 $ kg^–1^), given that 70% of the BAU production cost comes from ethylene (followed by Cl_2_, 20%; cooling, 7%; O_2_, 2%; and heating, 1%).^37^ As expected, the ideal routes show the best results, respectively, improvements of 56 and 55% for the ideal 1-step (0.36 $ kg^–1^) and ideal 2-step (0.37 $ kg^–1^). Regarding the real processes, the 2-step route (0.55 $ kg^–1^) reduces the production cost compared to the BAU (0.82 $ kg^–1^) by 32%, which is remarkable given the small amount of time this emerging technology has been under research. However, the 1-step route only shows an improvement of about 6% (0.77 $ kg^–1^) due to refrigeration playing a critical role in the process. Regardless of the small gain, these results display significant promise for the 1-step route, given that this is the first reported instance of this reaction system. While the 1-step real route cost contribution is led by cooling (33%), followed by Cl_2_ (29%), ethane (19%), O_2_ (8%), electricity (5%), and heating (2%); in the other ethane-based scenarios, the most important contribution to the cost is the Cl_2_ feed (44–52%), followed by ethane (24–26%), O_2_ (9–13%), cooling (4–14%), electricity (1–8%), and heating (1–2%).

**Figure 5:**
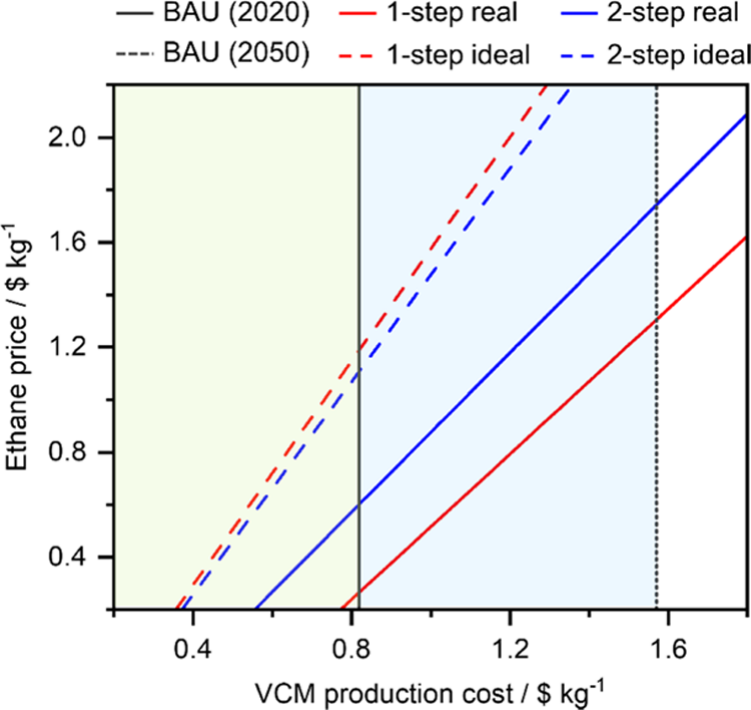
VCM production cost dependence on the ethane price of the five synthesis routes (BAU, 2-step, and 1-step, considering both the real and ideal scenarios). The ethylene price is fixed at 1.26 and 2.92 $ kg^–1^, considering a 132% increase by 2050 forecasted for oil.^37,38^ This is also accounted for in the BAU evaluation in 2020 and 2050. The price of utilities and other raw materials is considered constant. The economic advantage of ethane chlorination technologies over the BAU is highlighted: green for the current scenario and blue/green for the prospective scenario.

The ethane price is highly tied to the natural gas price, which has recently become highly volatile, mostly due to geopolitical factors. Accordingly, we next study its influence on the VCM production cost relative to the BAU (Figure 5). The 1-step and 2-step ideal ethane routes are quite robust against variations in the ethane prices, as these would need to increase six- and five-fold, respectively, for the BAU to outperform them. Furthermore, the 2-step real route would be cheaper than the BAU, even for a threefold increase in the ethane price. Finally, the 1-step real ethane route also outperforms the BAU at current prices, but in this case, price increases in ethane of 30% would suffice for both routes to become equally appealing, economically speaking. While these results already show the remarkable economic potential of the ethane-based routes, their financial advantage over the BAU could improve even further, considering that the oil price is projected to grow approximately 130% from 2020 to 2050.^38^ Considering this increase, the new price of ethylene in 2050 would ascend to 2.92 $ kg^–1^, which would lead to a VCM production cost of 1.57 $ kg^–1^. Under this scenario, the 1-step and 2-step ideal ethane routes would outperform the BAU even if ethane prices would increase ten-fold and 6 to 4 times for the 2-step and 1-step real scenarios. Overall, these are very promising results given the yet low-maturity level of the ethane-based routes compared to the long-time development of the BAU, which has already benefitted from learning curves and economies of scale.

## Conclusions

In this work, we evaluated the synthesis of VCM via two catalytic ethane chlorination technologies and compared them with the ethylene-based BAU using process simulation and LCA. Because the cost and footprint of ethane are substantially lower than those of ethylene, all of the emerging technologies have the potential to outperform the BAU. Specifically, at their current low-maturity level, both are already economically superior. However, the expected decarbonization of the power mix will render both technologies environmentally better, even at their current stage of development, and more so when assuming an ideal catalyst with full conversion and selectivity. Moreover, the economic and environmental appeal of the emerging routes is expected to improve in the mid-term when considering future cost and impact projections. Specifically, ethane chlorination catalytic technologies could halve the climate change impact of current ethylene-derived VCM synthesis. Besides, due to the low ethane price (6 times lower) relative to ethylene, whose price is expected to double in the future, the emerging technologies will have the potential to lead to a win–win scenario in which both environmental and economic criteria could be simultaneously improved through their adoption. This would avoid the need to define subsidies and enforce regulations to promote their deployment.

Going beyond VCM synthesis, we stress the importance of performing prospective LCAs to shed light on the future role of emerging technologies and evaluate their potential in the not-so-distant future. Specifically, from a practical side, our results suggest the need to revisit previous studies on emerging technologies that may have failed to provide a comprehensive picture of their true potential because they overlooked future defossilization efforts in sectors connected to chemicals production.

## Supporting Information Available

Experimental work (section A); design of the simulations (section B); additional results (section C); and the economic analysis parameters (section D) (PDF)

## Abbreviations

BAU: business-as-usual
CO_2_-eq: CO_2_ equivalent
EC: ethylene carbonate
EDC: 1,2-dichloroethane
LCA: life cycle assessment
LCI: life cycle inventory
LCIA: life cycle impact assessment
PVC: poly(vinyl chloride)
TCE: trichloroethane
VCM: vinyl chloride monomer

## References

[ref1] Lin R.; Amrute A. P.; Pérez-Ramírez J. Halogen-Mediated Conversion of Hydrocarbons to Commodities. Chem. Rev. 2017, 117, 4182–4247. 10.1021/acs.chemrev.6b00551. 28150944

[ref2] Geyer R.; Jambeck J. R.; Law K. L. Production, Use, and Fate of All Plastics Ever Made. Sci. Adv. 2017, 3, e1700782 10.1126/sciadv.1700782. 28776036 PMC5517107

[ref3] Vallette J. Chlorine and Building Materials: A Global Inventory of Production Technologies and Markets, 2019. https://healthybuilding.net/uploads/files/Chlorine%20&%20Building%20Materials%20Phase%201%20-%20v2.pdf.

[ref4] Ma H.; Wang Y.; Qi Y.; Rout K. R.; Chen D. Critical Review of Catalysis for Ethylene Oxychlorination. ACS Catal. 2020, 10, 9299–9319. 10.1021/acscatal.0c01698.

[ref5] Wernet G.; Bauer C.; Steubing B.; Reinhard J.; Moreno-Ruiz E.; Weidema B. The Ecoinvent Database Version 3 (Part I): Overview and Methodology. Int. J. Life Cycle Assess. 2016, 21, 1218–1230. 10.1007/s11367-016-1087-8.

[ref6] Benyahia F. The VCM Process Economics: Global and Raw Material Impacts, Proceedings of the 1st Annual Gas Processing Symposium; Elsevier, 2009.

[ref7] Carroll R. T.; De Witt E. W.; Trapasso L. E. Oxychlorination of Lower Alkanes. U.S. Patent. US3,173,9621965.

[ref8] Croce L. J.; Bajars L.; Gabliks M. Oxychlorination of Hydrocarbons in the Presence of Non-Halide Copper Containing Catalysts. U.S. Patent. US4,025,4611975.

[ref9] DeRosa S. E.; Allen D. T. Impact of Natural Gas and Natural Gas Liquids Supplies on the United States Chemical Manufacturing Industry: Production Cost Effects and Identification of Bottleneck Intermediates. ACS Sustainable Chem. Eng. 2015, 3, 451–459. 10.1021/sc500649k.

[ref10] Chen Q.; Dunn J. B.; Allen D. T. Mapping Greenhouse Gas Emissions of the U.S. Chemical Manufacturing Industry: The Effect of Feedstock Sourcing and Upstream Emissions Allocation. ACS Sustainable Chem. Eng. 2022, 10, 5932–5938. 10.1021/acssuschemeng.2c00295.

[ref11] Scharfe M.; Zichittella G.; Kondratenko V. A.; Kondratenko E. V.; López N.; Pérez-Ramírez J. Mechanistic Origin of the Diverging Selectivity Patterns in Catalyzed Ethane and Ethene Oxychlorination. J. Catal. 2019, 377, 233–244. 10.1016/j.jcat.2019.07.021.

[ref12] Zhang H.-M.; Fan Q.-Y.; Zhang Q.-H.; Kang J.-C.; Wang Y.; Cheng J. Understanding Catalytic Mechanisms of Alkane Oxychlorination from the Perspective of Energy Levels. J. Phys. Chem. C 2020, 124, 6070–6077. 10.1021/acs.jpcc.9b10464.

[ref13] Hickman D. A.; Jones M. E.; Jovanovic Z. R.; Olken M. M.; Podkolzin S. G.; Stangland E. E.; Thompson R. K. Reactor Scale-up for Fluidized Bed Conversion of Ethane to Vinyl Chloride. Ind. Eng. Chem. Res. 2010, 49, 10674–10681. 10.1021/ie100423z.

[ref14] W D Clarke W. D.; Haymon T. D.; Henley J. P.; Hickman D. A.; Jones M. E.; Miller M. C.; Morris T. E.; Reed D. J.; Smason L. J.; Smith S. A. Process for Vinyl Chloride Manufacture from Ethane and Ethylene with Air Feed and Altermative HCl Processing Methods. U.S. Patent. US152,9292004.

[ref15] Zichittella G.; Pérez-Ramírez J. Ethane-Based Catalytic Process for Vinyl Chloride Manufacture. Angew. Chem. 2021, 133, 24291–24297. 10.1002/ange.202105851. 34288317

[ref16] Economides M. J.; Wood D. A. The State of Natural Gas. J. Nat. Gas Sci. Eng. 2009, 1, 1–13. 10.1016/j.jngse.2009.03.005.

[ref17] Evans R. B. Chlorine: State of the Art. Lung 2005, 183, 151–167. 10.1007/s00408-004-2530-3. 16078037

[ref18] Pérez-Fortes M.; Mian A.; Srikanth S.; Wang L.; Diethelm S.; Varkaraki E.; Mirabelli I.; Makkus R.; Schoon R.; Maréchal F.; Van Herle J. Design of a Pilot SOFC System for the Combined Production of Hydrogen and Electricity under Refueling Station Requirements. Fuel Cells 2019, 19, 389–407. 10.1002/fuce.201800200. 31680792 PMC6813630

[ref19] Zichittella G.; Ceruti A.; Guillén-Gosálbez G.; Pérez-Ramírez J. Catalyst: A step Forward for PVC Manufacture from Natural Gas. Chem 2022, 8, 883–885. 10.1016/j.chempr.2022.02.012.

[ref20] Roh K.; Bardow A.; Bongartz D.; Burre J.; Chung W.; Deutz S.; Han D.; Heßelmann M.; Kohlhaas Y.; König A.; Lee J. S.; Meys R.; Völker S.; Wessling M.; Lee J. H.; Mitsos A. Early-Stage Evaluation of Emerging CO_2_ Utilization Technologies at Low Technology Readiness Levels. Green Chem. 2020, 22, 3842–3859. 10.1039/C9GC04440J.

[ref21] Piccinno F.; Hischier R.; Seeger S.; Som C. From Laboratory to Industrial Scale: a Scale-up Framework for Chemical Processes in Life Cycle Assessment Studies. J. Cleaner Prod. 2016, 135, 1085–1097. 10.1016/j.jclepro.2016.06.164.

[ref22] Sacchi R.; Terlouw T.; Siala K.; Dirnaichner A.; Bauer C.; Cox B.; Mutel C.; Daioglou V.; Luderer G. PRospective EnvironMental Impact asSEment (Premise): A Streamlined Approach to Producing Databases for Prospective Life Cycle Assessment Using Integrated Assessment Models. Renewable Sustainable Energy Rev. 2022, 160, 112311 10.1016/j.rser.2022.112311.

[ref23] Mutel C. Brightway: An Open Source Framework for Life Cycle Assessment. J. Open Source Software 2017, 2, 236 10.21105/joss.00236.

[ref24] Stehfest E.; van Vuuren D.; Kram T.; Bouwman L.; Alkemade R.; Bakkenes M.; Biemans H.; Bouwman A.; den Elzen M.; Janse J.; Lucas P.; van Minnen J.; Müller C.; Prins A. G. Integrated Assessment of Global Environmental Change with IMAGE 3.0 – Model Description and Policy Applications; Netherlands Environmental Assessment Agency, 2014; pp 1–366.

[ref25] Lakshmanan A.; Rooney W. C.; Biegler L. T. A Case Study for Reactor Network Synthesis: the Vinyl Chloride Process. Comput. Chem. Eng. 1999, 23, 479–495. 10.1016/S0098-1354(98)00287-7.

[ref26] Dreher E.-L.; Beutel K. K.; Myers J. D.; Lübbe T.; Krieger S.; Pottenger L. H. Chloroethanes and Chloroethylenes. Ullmann’s Encycl. Ind. Chem. 2014, 1, 1–81. 10.1002/14356007.o06_o01.pub2.

[ref27] ISO – ISO 14040:2006 – Environmental management – Life cycle assessment – Principles and framework.

[ref28] Huijbregts M. A.; Steinmann Z. J.; Elshout P. M. F.; Stam G.; Verones F.; Vieira M. D.; Hollander A.; Zijp M.; van Zelm R. ReCiPe 2016 v1.1, RIVM Rep. 2016-0104. 2017, www.rivm.nl/en.

[ref29] Medrano-García J. D.; Javaloyes-Antón J.; Vázquez D.; Ruiz-Femenia R.; Caballero J. A. Alternative Carbon Dioxide Utilization in Dimethyl Carbonate Synthesis and Comparison with Current Technologies. J. CO_2_ Util. 2021, 45, 101436 10.1016/j.jcou.2021.101436.

[ref30] González-Garay A.; Frei M. S.; Al-Qahtani A.; Mondelli C.; Guillén-Gosálbez G.; Pérez-Ramírez J. Plant-to-Planet Analysis of CO_2_-Based Methanol Processes. Energy Environ. Sci. 2019, 12, 3425–3436. 10.1039/C9EE01673B.

[ref31] Galán-Martín Á.; Tulus V.; Díaz I.; Pozo C.; Pérez-Ramírez J.; Guillén-Gosálbez G. Sustainability Footprints of a Renewable Carbon Transition for the Petrochemical Sector Within Planetary Boundaries. One Earth 2021, 4, 565–583. 10.1016/j.oneear.2021.04.001.

[ref32] Sicotte D. M. From Cheap Ethane to a Plastic Planet: Regulating an Industrial Global Production Network. Energy Res. Soc. Sci. 2020, 66, 101479 10.1016/j.erss.2020.101479.

[ref33] Potrč S.; Čuček L.; Martin M.; Kravanja Z. Sustainable Renewable Energy Supply Networks Optimization – The Gradual Transition to a Renewable Energy System Within the European Union by 2050. Renewable Sustainable Energy Rev. 2021, 146, 111186 10.1016/j.rser.2021.111186.

[ref34] Ioannou I.; Javaloyes-Antón J.; Caballero J. A.; Guillén-Gosálbez G. Economic and Environmental Performance of an Integrated CO_2_ Refinery. ACS Sustainable Chem. Eng. 2023, 11, 1949–1961. 10.1021/acssuschemeng.2c06724. 36778522 PMC9906749

[ref35] Luyben W. L. Estimating Refrigeration Costs at Cryogenic Temperatures. Comput. Chem. Eng. 2017, 103, 144–150. 10.1016/j.compchemeng.2017.03.013.

[ref36] Messineo A.; Volpe R.; Marvuglia A. Ligno-Cellulosic Biomass Exploitation for Power Generation: A Case Study in Sicily. Energy 2012, 45, 613–625. 10.1016/j.energy.2012.07.036.

[ref37] Dutta A.; Karimi I. A.; Farooq S. Technoeconomic Perspective on Natural Gas Liquids and Methanol as Potential Feedstocks for Producing Olefins. Ind. Eng. Chem. Res. 2019, 58, 963–972. 10.1021/acs.iecr.8b05277.

[ref38] U.S. Energy Information Administration Total Energy Supply, Disposition, and Price Summary, Annual Energy Outlook, 2021. https://www.eia.gov/outlooks/aeo/pdf/appa.pdf. accessed April 2023.

