# Supplementary material for: Economic and Environmental Competitiveness of Ethane-Based Technologies for Vinyl Chloride Synthesis
Source: ACS Sustain Chem Eng. 2023 Aug 23;11(35):13062–9. doi: 10.1021/acssuschemeng.3c03006 (PMC10481392; doi:10.1021/acssuschemeng.3c03006)
Supplement: Supplementary file 1 — sc3c03006_si_001.pdf [file sc3c03006_si_001.pdf]

Economic and Environmental Competitiveness of Ethane-Based Technologies for Vinyl Chloride Synthesis

Juan D. Medrano-García,[†] *Vera Giulimondi,*[†] *Amedeo Ceruti,*[†] *Guido Zichittella,*[†] *Javier Pérez-Ramírez,*[*,†] *and Gonzalo Guillén-Gosálbez*[*,†]

[†] Institute for Chemical and Bioengineering, Department of Chemistry and Applied Biosciences, ETH Zurich, Vladimir-Prelog-Weg 1, 8093 Zurich, Switzerland.

* Corresponding authors.

E-mail addresses: jpr@chem.ethz.ch, gonzalo.guillen.gosalbez@chem.ethz.ch.

This document includes additional material to the content presented in the main article. Here we describe the experimental procedure, the process simulations, the computation of the life cycle inventories, further environmental results, and the data used in the economic analysis.

Number of pages: 29

Number of figures: 10

Number of tables: 19

Table of contents

A. Catalyst testing..3

B. Process simulations 5

B.1. Ethylene chlorination and oxychlorination to EDC and cracking (BAU) 6

B.2. Ethane chlorination to EDC and cracking (2-step real) 9

B.3. Ideal ethane chlorination to EDC and cracking (2-step ideal) 11

B.4. Direct ethane chlorination to VCM (1-step real) 12

B.5. Ideal direct ethane chlorination to VCM (1-step ideal)..14

C. Life cycle assessment (LCA) 16

C.1. Life cycle inventories (LCIs) 16

C.2. Damage assessment...23

D. Economic assessment...25

D.1. Variable operating costs..25

D.2. Capital investment costs..26

E. References 28

A. Catalyst testing

The chlorination of ethane was evaluated at atmospheric pressure in a continuous-flow fixed-bed micro-reactor, described elsewhere,[1] over commercial γ-Al_2O_3 (Alfa Aesar), calcined in static air at 350 °C prior to its use in the catalytic test. Briefly, the gases C_2H_6 (PanGas, purity 3.5), Cl_2 (PanGas, purity 2.8, anhydrous), Ar (PanGas, purity 5.0; internal standard), and He (PanGas, purity 5.0; carrier gas) were fed using digital mass-flow controllers (Bronkhorst®) to a mixing unit. A quartz reactor (internal diameter, d_i = 8 mm) was loaded with the catalyst (mass, m_{cat} = 0.1 g; particle size, d_p = 0.2-0.4 mm), and placed in an electrical oven. A K-type thermocouple fixed in a co-axial quartz thermowell with the tip positioned in the center of the catalyst bed (volume, V_{bed} = 0.15 cm^3) was used to monitor the temperature during the reaction. Prior to testing, the bed was heated in He to the desired temperature (T = 325 °C) and allowed to stabilize for at least 30 min before the reaction mixture was fed (volumetric flow, F_T = 20 cm^3 min^{-1}, C_2H_6:Cl_2:Ar:He = 6:6:4.5:83.5) at a gas-hourly space velocity ($GHSV = F_T:V_{bed}$) of 8000 h^{-1}. All flow units correspond to standard temperature and pressure (STP) conditions, *i.e.*, 0 °C and 1 bar. Down-stream heat tracing at 120 °C prevented the condensation of liquefiable unconverted reactants and products. The effluent gas stream was passed through impinging bottles, containing an aqueous 1 M NaOH solution for neutralization prior to its release in the ventilation system. Carbon-containing compounds and Ar were quantified on-line *via* a gas chromatograph equipped with a GS-Carbon PLOT column coupled to a mass spectrometer (GC MS, Agilent GC 6890, Agilent MSD 5973N). The carbon containing compounds are: ethane (C_2H_6), ethylene (C_2H_4), ethyl chloride (C_2H_5Cl, EC), vinyl chloride (C_2H_3Cl, VCM), 1,2-dichloroethane (1,2-$C_2H_4Cl_2$, EDC), 1,1-dichloroethane (1,1-$C_2H_4Cl_2$) and 1,1,2-trichloroethane (1,1,2-$C_2H_3Cl_3$., TCE).

The conversion of C_2H_6, $X_{C_2H_6}$, the selectivity, S_j, the yield, Y_j, of product *j* (*j*: C_2H_4, C_2H_5Cl, C_2H_3Cl, 1,2-$C_2H_4Cl_2$, 1,1-$C_2H_4Cl_2$, and 1,1,2-$C_2H_3Cl_3$), the error of the carbon mass balance, ε_C, were calculated using Eqs. (1)-(4), respectively, in which $n_{C_2H_6}^{inlet}$ and $n_{C_2H_6}^{outlet}$ are the molar flows of C_2H_6 at the reactor inlet and outlet, respectively, n_j^{outlet} is the molar flow of product *j* at the reactor outlet, and N_{C,C_2H_6} and $N_{C,j}$ are the number of carbon atoms in C_2H_6 and in product *j*, respectively.

$$X_{C_2H_6} = \frac{n_{C_2H_6}^{inlet} - n_{C_2H_6}^{outlet}}{n_{C_2H_6}^{inlet}} \times 100, \% \quad (1)$$

$$S_j = \frac{n_j^{\mathrm{outlet}} - N_{\mathrm{C},j}}{\left(n_{\mathrm{C_2H_6}}^{\mathrm{inlet}} - n_{\mathrm{C_2H_6}}^{\mathrm{outlet}}\right) \times N_{\mathrm{C,C_2H_6}}} \times 100, \% \tag{2}$$

$$Y_j = \frac{X_{\mathrm{C_2H_6}} \times S_j}{100}, \% \tag{3}$$

$$\varepsilon_{\mathrm{C}} = \frac{n_{\mathrm{C_2H_6}}^{\mathrm{inlet}} \times N_{\mathrm{C,C_2H_6}} - \left(n_{\mathrm{C_2H_6}}^{\mathrm{outlet}} \times N_{\mathrm{C,C_2H_6}} + \sum n_j^{\mathrm{outlet}} \times N_{\mathrm{C},j}\right)}{n_{\mathrm{C_2H_6}}^{\mathrm{inlet}} \times N_{\mathrm{C,C_2H_6}}}, \% \tag{4}$$

The error of the carbon mass balance was less than 5% in all experiments, i.e., the carbon mass balance was closed at ≥95%.

B. Process simulations

We developed a range of simulations differing in the underlying assumptions. We describe next each of these simulations in detail. We use the NRTL-RK thermodynamic package in Aspen Plus v12 to model the system as suggested by the literature, including a set of NRTL parameters for the azeotropic pair EDC and water (**Table S.1**).[2,3] In the case of the columns employed for HCl recovery by absorption, we use the ENRTL-RK to handle the presence and effect of electrolytes in the unit operation. Unless stated otherwise, streams are fed to distillation columns at their dew point (vapor streams) or bubble point (liquid streams) and reactors are operated isothermally. Finally, we compute the minimum utility consumption with Aspen Energy Analyzer. The inventories resulting from the material and energy flows of the simulations are presented in **Section C**.

Table S.1. NRTL interaction parameters for the pair EDC (component *i*) and water (component *j*).[2]

Parameter	Value
a_{ij}	$2.372\cdot10^{2}$
a_{ji}	$9.365\cdot10^{1}$
b_{ij}	$-8.794\cdot10^{3}$
b_{ji}	$-4.071\cdot10^{3}$
c_{ij}	$2.000\cdot10^{-1}$
d_{ij}	-
e_{ij}	$-3.594\cdot10^{1}$
e_{ji}	$-1.320\cdot10^{1}$
f_{ij}	-
f_{ji}	-

B.1. Ethylene chlorination and oxychlorination to EDC and cracking (BAU)

The business-as-usual (BAU) simulation (**Figure S.1**) considers the state-of-the-art VCM production from ethylene chlorination and oxychlorination with subsequent EDC cracking.[4]

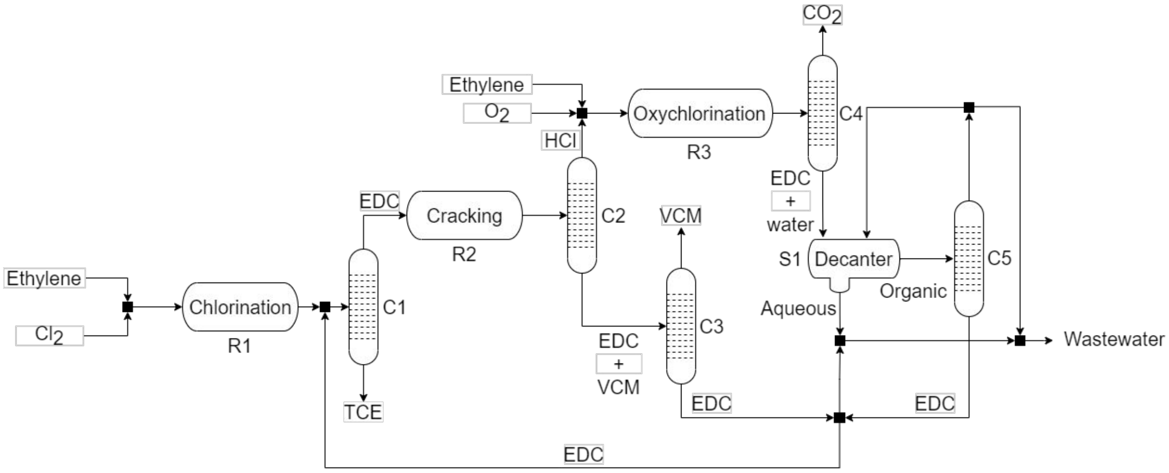

Figure S.1. Ethylene chlorination and oxychlorination to EDC and cracking (BAU scenario) simplified flowsheet.

An equimolar mixture of ethylene and Cl_2 at standard conditions is heated up to 60 ºC and sent to the chlorination reactor R1.[5] Here, EDC is formed with high ethylene conversion (97.8%) and selectivity (98.9%).[5] Additionally, TCE is obtained as an undesired byproduct (1.09% ethylene conversion) (Eqs.(5),(6)).

$$C_2H_4 + Cl_2 \rightarrow C_2H_4Cl_2 \text{ (EDC)} \tag{5}$$

$$C_2H_4 + 2Cl_2 \rightarrow C_2H_3Cl_3 \text{ (TCE)} + HCl \tag{6}$$

The effluent of the reactor is heated up to its bubble point and enters distillation column C1 where TCE is recovered as the bottom product. The distillate is then compressed to 20 bar, heated up to 500 ºC and sent to the cracking reactor R2. Here, EDC is cracked into VCM and HCl with 60 % conversion (Eq.(7)).[5]

$$C_2H_4Cl_2 \text{ (EDC)} \leftrightarrow C_2H_3Cl \text{ (VCM)} + HCl \tag{7}$$

The mixture is depressurized to 12 bar, cooled down to its dew point and sent to distillation column C2. Unreacted EDC and VCM are recovered as the bottom product, while HCl is obtained as the distillate. The HCl stream is then mixed with ethylene and O_2 (previously compressed to 5.3 bar) in a 2:1:0.5 molar ratio, heated up to 193 ºC and sent to the oxychlorination reactor R3. Here, ethylene, HCl and O_2 are converted into EDC and water, with

TCE formation and ethylene combustion as other notable secondary reactions (Eqs.(8)-(10)). The ethylene conversions for these reactions are 99.8, 0.12 and 0.03%, respectively.[5]

$$C_2H_4 + HCl + 0.5O_2 \rightarrow C_2H_4Cl_2\ (EDC) + H_2O \tag{8}$$

$$C_2H_4 + 3HCl + O_2 \rightarrow C_2H_3Cl_3\ (TCE) + 2H_2O \tag{9}$$

$$C_2H_4 + 3O_2 \rightarrow 2CO_2 + 2H_2O \tag{10}$$

After the reaction, the mixture is cooled down to 30 ºC and the small gas fraction (CO_2, Cl_2 and unreacted HCl, O_2 and ethylene) is removed with an ideal component splitter (C4) to avoid convergence issues. The rest is sent to the decanter S1 where the aqueous phase is recovered as wastewater and the organic phase is sent to stripper column C5. In this column, dried EDC is obtained as the bottom product, which is recycled back to C1, while the top product is returned to the decanter after a small purge. The liquid product of C2, mainly containing EDC and VCM, is sent to distillation column C3 where VCM 99.4 wt.% is obtained as the top product. The tails of the column contain mostly EDC that is recycled back to C1. The column specifications of the simulation are shown in **Table S.2, Table S.3** and **Table S.4.**

The chlorinated hydrocarbons contained in the wastewater (**Figure S.2**) are assumed to be recovered by flocculation (S1) and ideally combusted in a furnace (F1) with stoichiometric air to produce HCl, CO_2 and water (Eqs.(11)-(13)).[6] By washing this flue gas with water in an absorber column (A1) at 1 bar and 40 ºC, HCl 30 wt.% is recovered as a byproduct.

$$C_2H_3Cl\ (VCM) + 2.5O_2 \rightarrow HCl + 2CO_2 + H_2O \tag{11}$$

$$C_2H_4Cl_2\ (EDC) + 2.5O_2 \rightarrow 2HCl + 2CO_2 + H_2O \tag{12}$$

$$C_2H_3Cl_3\ (TCE) + 2O_2 \rightarrow 3HCl + 2CO_2 \tag{13}$$

The recovered HCl 30 wt.% is sent to the Deacon oxidation process (**Figure S.3**). Here, a stream with a 4:1 molar ratio of HCl and O_2 at atmospheric pressure is heated up to 350 ºC. The mixture enters the oxidation reactor R1, where the oxidation reaction (Eq.(14)) takes place at an 85% HCl conversion.[7,8] After the reactor, the products are separated using an ideal component splitter (C1+C2+C3). Cl_2 is obtained as the main product while unreacted HCl and O_2 are recycled back to the reactor inlet. Finally, water is discarded as a waste stream.

$$4HCl + O_2 \leftrightarrow 2Cl_2 + 2H_2O \tag{14}$$

Table S.2. Ethylene chlorination and oxychlorination to EDC and cracking (BAU scenario) separation design specifications (C1-C3).[3,4]

Column	Light key recovery in distillate [%]	Heavy key recovery in distillate [%]	Reflux ratio*	Condenser
C1	$9.99\cdot10^{-1}$ (EDC)	$1.00\cdot10^{-3}$ (TCE)	1.30 R_{min}	Total
C2	$9.99\cdot10^{-1}$ (HCl)	$2.50\cdot10^{-2}$ (VCM)	1.50 R_{min}	Partial
C3	$9.99\cdot10^{-1}$ (VCM)	$1.00\cdot10^{-3}$ (EDC)	1.40 R_{min}	Total

*R_{min} stands for minimum reflux ratio.

Table S.3. Ethylene chlorination and oxychlorination to EDC and cracking (BAU scenario) separation design specifications (C4).[3,4]

Column	Component	Distillate split fraction
C4	Cl_2	1
	Ethylene	1
	VCM	0
	HCl	1
	O_2	1
	H_2O	0
	EDC	0
	TCE	0
	CO_2	1

Table S.4. Ethylene chlorination and oxychlorination to EDC and cracking (BAU scenario) separation design specifications (C5).[2]

Column	Stages	Feed stage	Boilup ratio	Condenser
C5	30	1	1.20	None

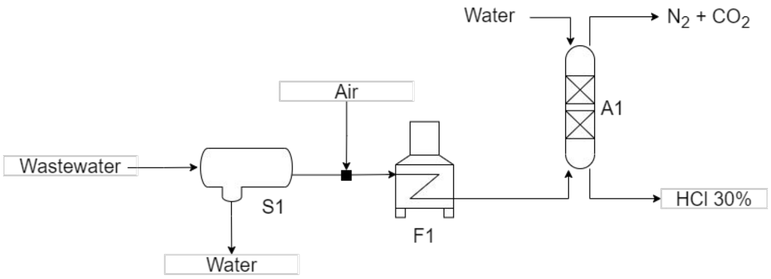

Figure S.2. General wastewater treatment simplified flowsheet.

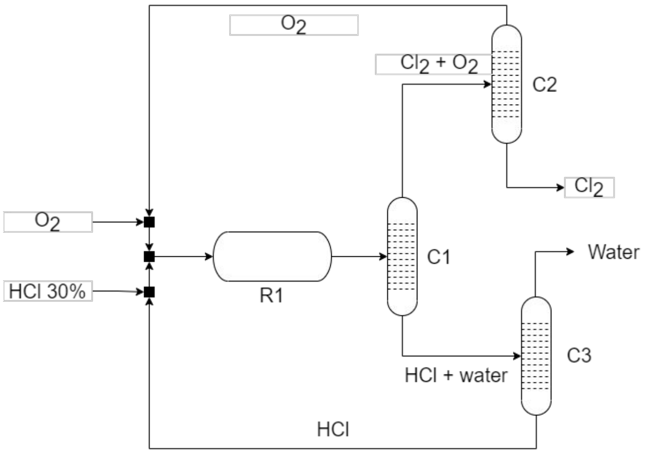

Figure S.3. General Deacon oxidation process simplified flowsheet.

B.2. Ethane chlorination to EDC and cracking (2-step real)

The 2-step ethane real route (**Figure S.4**) is simulated using experimental data, reported elsewhere,[1] to model the chlorination reactor, while the cracking reactor is assumed to perform as in the BAU.

An atmospheric pressure 1:6 molar ratio of ethane and Cl_2 stream (22.02 $kg \cdot s^{-1}$) is heated up to 300 ºC and enters the ethane chlorination reactor R1. Here, the two reagents react isothermally to form EDC, EC and TCE with 75, 23 and 2% product yields, respectively (Eqs.(15)-(17)):

$$C_2H_6 + 2Cl_2 \rightarrow C_2H_4Cl_2 \text{ (EDC)} + 2HCl \quad (15)$$

$$C_2H_6 + Cl_2 \rightarrow C_2H_5Cl \text{ (EC)} + HCl \quad (16)$$

$$C_2H_6 + 3Cl_2 \rightarrow C_2H_3Cl_3 \text{ (TCE)} + 3HCl \quad (17)$$

The reaction products are cooled down to 26 ºC and compressed to 10 bar before entering the first distillation column (C1), where unreacted Cl_2 and HCl leave as the distillate. The gases are then sent to an absorber (A1) where HCl is removed with water at 1 bar and 40 ºC producing

HCl 30 wt.% is sent to the Deacon process section. The cleaned Cl_2 stream is finally recycled back to the reactor inlet after removing the water (C6). The bottom product of C1 is sent to a second distillation column (C2) where EC is recovered as the top product. The EDC bottom product is compressed to 20 bar and heated up to 500 ºC before entering the cracking reactor R2. After the reaction, the products enter distillation column C3 at 12 bar and 11 ºC. The distillate consists of HCl which is sent to the absorber. The bottom product mainly contains EDC and VCM, which enter distillation column C4 at 5 bar and 57 ºC. Here, VCM 99.6 wt.% is recovered as the top product, while the bottom EDC-rich stream is depressurized to 3 bar, cooled down to 121 ºC and sent to distillation column C5, where it is purified and recycled back to the cracking reactor. The wastewater along with the purge is sent to the organochlorine waste section. The column specifications of the simulation are shown in **Table S.5**.

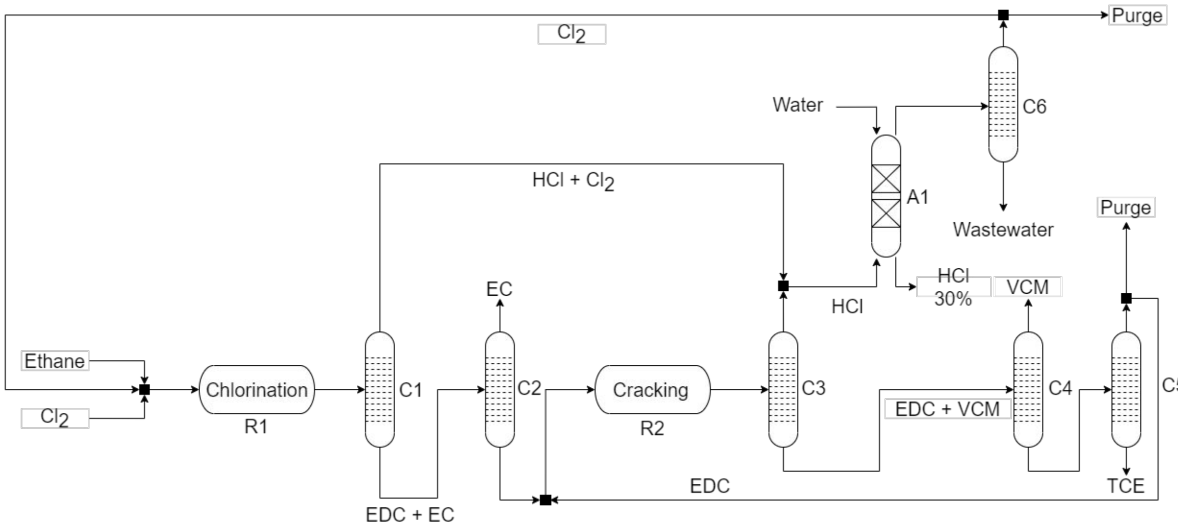

Figure S.2. Ethane chlorination to EDC and cracking (2-step real scenario) simplified flowsheet.

Table S.5. Ethane chlorination to EDC and cracking (2-step real scenario) separation design specifications (C1-C5).

Column	Light key recovery in distillate [%]	Heavy key recovery in distillate [%]	Reflux ratio*	Condenser
C1	$9.99\cdot10^{-1}$ (Cl_2)	$1.00\cdot10^{-4}$ (EC)	1.30 R_{min}	Partial
C2	$9.99\cdot10^{-1}$ (EC)	$1.00\cdot10^{-4}$ (EDC)	1.30 R_{min}	Total
C3	$9.95\cdot10^{-1}$ (HCl)	$2.50\cdot10^{-2}$ (VCM)	1.50 R_{min}	Total
C4	$9.96\cdot10^{-1}$ (VCM)	$1.00\cdot10^{-3}$ (VCM)	1.40 R_{min}	Total
C5	$9.97\cdot10^{-1}$ (EDC)	$1.00\cdot10^{-4}$ (TCE)	1.40 R_{min}	Total

*R_{min} stands for minimum reflux ratio

B.3. Ideal ethane chlorination to EDC and cracking (2-step ideal)

In the 2-step ethane ideal route (**Figure S.5**), ethane and Cl_2 (1:2 molar ratio) at 1 bar and 20 ºC enter the process and are heated up to 300 ºC before being fed to the chlorination reactor. Here, the EDC formation reaction (Eq.(15)) with a 100% conversion of ethane is considered to take place isothermally. The mixture is then compressed to 21 bar in three stages with intercooling before entering the first separation section. After a flash separator working at 35 ºC, the liquid stream is depressurized to 12 bar and sent to a second flash separator. The liquid phase continues to an ideal component splitter to further remove the impurities (HCl and Cl_2) and separate pure EDC as the liquid stream. This EDC is recompressed to 18 bar and heated up to 500 ºC before entering the cracking reactor (Eq.(8)). After the reaction, the mixture is quenched to 157 ºC and flashed. The vapor phase is sent to a furnace, while the liquid enters the second separation section. Here, its temperature and pressure are adjusted to 12 bar and 130ºC before entering the first distillation column (C1) where HCl is removed as the top product. This HCl is mixed with the vapor streams of the first separation sections and liquid water, producing HCl 30 wt.%. The liquid product of C1 is then fed at 5 bar and 67 ºC to a second distillation column (C2). Here, unreacted EDC is recovered as the bottom product and sent to the third separation section. The distillate, with 98.74 wt.% VCM, enters the third and last distillation column (C3) where it is purified to 99.98%. In the last separation section, the heavy components of the raw EDC stream are removed with an ideal component splitter and sent to the furnace, while the EDC with traces of Cl_2, HCl and VCM is recycled back to the cracking reactor. The combustion

gases of the furnace are then sent to the Deacon oxidation process to recover Cl_2 from HCl. The column specifications of the simulation are shown in **Table S.6**.

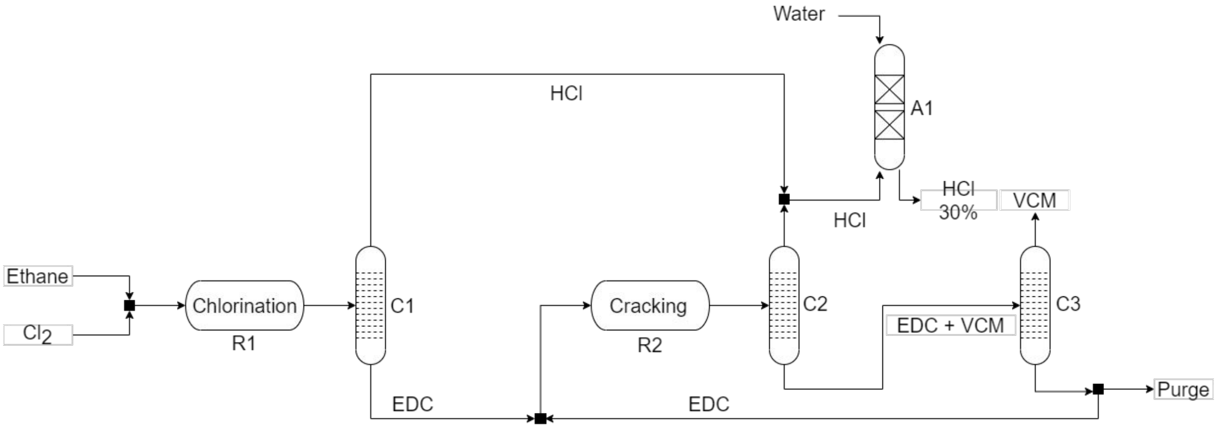

Figure S.3. Ideal ethane chlorination to EDC and cracking (2-step ideal scenario) simplified flowsheet.

Table S.6. Ideal ethane chlorination to EDC and cracking (2-step ideal scenario) separation design specifications (C1-C5).

Column	Light key recovery in distillate [%]	Heavy key recovery in distillate [%]	Reflux ratio*	Condenser
C1	$9.99 \cdot 10^{-1}$ (Cl_2)	$1.00 \cdot 10^{-4}$ (EC)	1.30 R_{min}	Partial
C2	$9.95 \cdot 10^{-1}$ (HCl)	$2.50 \cdot 10^{-2}$ (VCM)	1.50 R_{min}	Total
C3	$9.96 \cdot 10^{-1}$ (VCM)	$1.00 \cdot 10^{-3}$ (VCM)	1.40 R_{min}	Total

*R_{min} stands for minimum reflux ratio

B.4. Direct ethane chlorination to VCM (1-step real)

The 1-step real route (**Figure S.6**) is based on experimental data acquired as described in **Section A**, where ethane and Cl_2 (1:1 molar ratio) at 1 bar and 325 ºC enter the chlorination reactor to yield EDC, ethylene, EC, 1,1-dichloroethane, VCM (with 1.60, 7.60, 1.20, 4.40 and 25.2% product yields, respectively) and HCl according to Eqs.(15)-(16),(18)-(20):

$$C_2H_6 + Cl_2 \rightarrow C_2H_4 \text{ (ethylene)} + 2HCl \quad (18)$$

$$C_2H_6 + 2Cl_2 \rightarrow C_2H_4Cl_2 \text{ (1,1-dichloroethane)} + 2HCl \quad (19)$$

$$C_2H_6 + 2Cl_2 \rightarrow C_2H_3Cl \text{ (VCM)} + 3HCl \quad (20)$$

After the reaction, the mixture is cooled down to saturation temperature and compressed to 12 bar. The stream is cooled down to saturation once more and enters the first distillation column (C1) where ethane, ethylene, Cl_2 and HCl (vapor) are separated from VCM, EDC, ethyl chloride and 1,1-dichloroethane (liquid). The vapor stream is then washed with water in an absorber to remove HCl while producing HCl 30 wt.%. The rest of the water is removed in a flash separator followed by an ideal component splitter. The stream then is fed to a cryogenic distillation (–56 ºC, 1 bar) column that recovers ethylene as the distillate. The ethane and Cl_2 mixture is then recycled back to the chlorination reactor after a 0.1% purge. The liquid product of C1 enters a second distillation column (C2) where VCM is obtained as the main product in the distillate. The liquid is compressed to 20 bar and fed to a third column (C3) to separate EDC as the bottom product. Finally, the distillate of C3 is fed to the last distillation column (C4) where EC and 1,1-dichloroethane are retrieved as the top and bottom products, respectively. The column specifications of the simulation are shown in **Table S.7**.

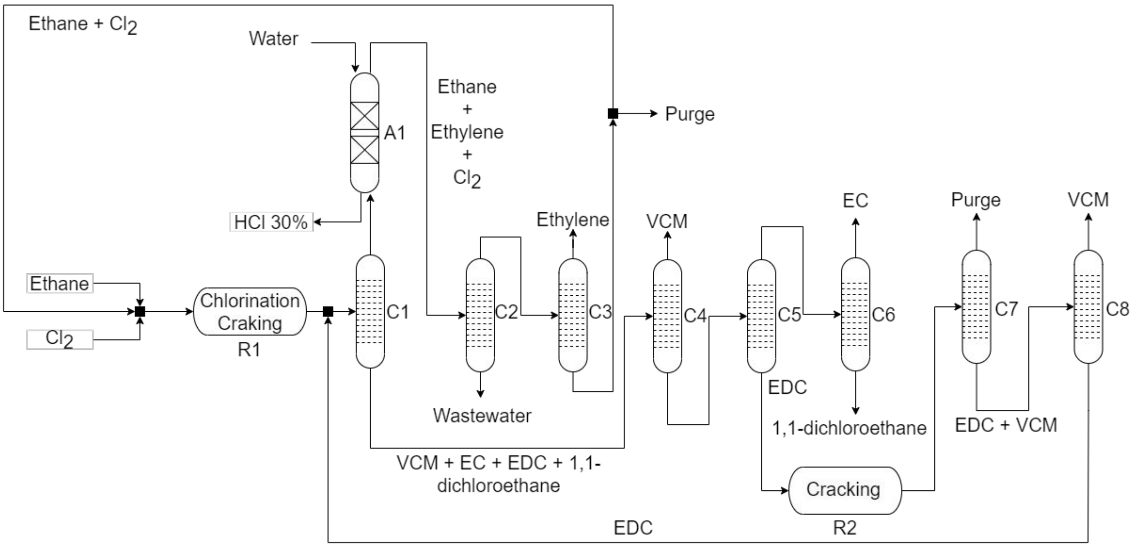

Figure S.4. Direct ethane chlorination to VCM (1-step real scenario) simplified flowsheet.

Table S.7. Direct ethane chlorination to VCM (1-step real scenario) separation design specifications (C1-C5).

Column	Light key recovery in distillate [%]	Heavy key recovery in distillate [%]	Reflux ratio*	Condenser
C1	$9.999 \cdot 10^{-1}$ (Cl_2)	$1.00 \cdot 10^{-4}$ (VCM)	1.30 R_{min}	Partial
C2	-	-	-	-
C3	$9.999 \cdot 10^{-1}$ (Ethylene)	$1.00 \cdot 10^{-4}$ (Ethane)	1.30 R_{min}	Partial
C4	$9.990 \cdot 10^{-1}$ (VCM)	$1.00 \cdot 10^{-3}$ (EC)	1.30 R_{min}	Total
C5	$9.990 \cdot 10^{-1}$ (1,1-dichloroethane)	$1.00 \cdot 10^{-3}$ (EDC)	1.30 R_{min}	Total
C6	$9.990 \cdot 10^{-1}$ (EC)	$1.00 \cdot 10^{-3}$ (1,1-dichloroethane)	1.30 R_{min}	Total
C7	$9.950 \cdot 10^{-1}$ (HCl)	$2.50 \cdot 10^{-3}$ (VCM)	1.50 R_{min}	Total
C8	$9.960 \cdot 10^{-1}$ (VCM)	$1.00 \cdot 10^{-3}$ (EDC)	1.40 R_{min}	Total

*R_{min} stands for minimum reflux ratio

B.5. Ideal direct ethane chlorination to VCM (1-step ideal)

In order to model the ideal version of the 1-step route (**Figure S.7**), we consider the same reaction conditions, but with stoichiometric feedstock according to Eq.(17), which is the only reaction taking place at 100% ethane conversion. The reaction products are cooled down to 40 ºC and compressed to 12 bar. They then enter distillation column C1 where VCM is recovered as the bottom product and HCl is obtained as the distillate. The HCl stream is treated with water (A1) to produce HCl 30 wt.%. The liquid stream is sent to the Deacon oxidation process to recover Cl_2, which is sent back to the reactor feedstock. The column specifications of the simulation are shown in **Table S.8**.

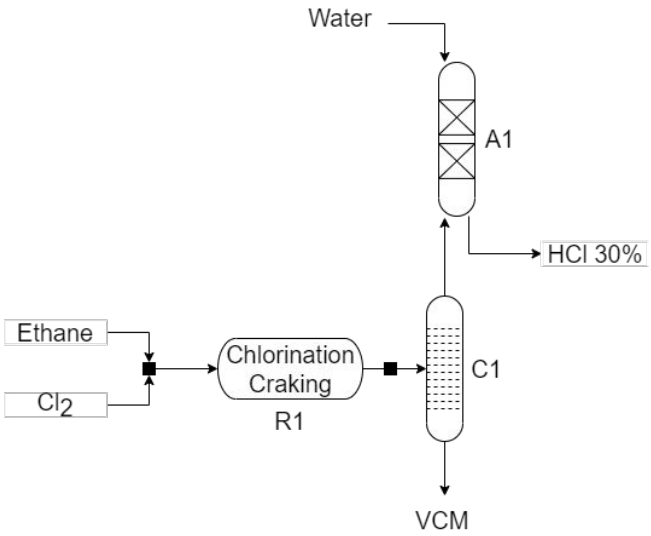

Figure S.5. Ideal direct ethane chlorination to VCM (1-step ideal scenario) simplified flowsheet.

Table S.8. Ideal direct ethane chlorination to VCM (1-step ideal scenario) separation design specifications (C1-C5).

Column	Light key recovery in distillate [%]	Heavy key recovery in distillate [%]	Reflux ratio*	Condenser
C1	$9.99 \cdot 10^{-1}$ (HCl)	$1.00 \cdot 10^{-3}$ (VCM)	1.30 R_{min}	Total

*R_{min} stands for minimum reflux ratio

C. Life cycle assessment (LCA)

In this section, we present the main results of the simulations as the net material and energy streams per functional unit, including the five VCM production scenarios, the Deacon process and the refrigeration inventories used for the environmental and economic analyses. Then, we show the damage assessment results (ReCiPe 2016 v1.1 endpoints) for all the studied scenarios.[9]

C.1. Life cycle inventories (LCIs)

In this section, we present the main results of the simulations (net material and energy streams per kg of VCM) in the form of LCIs.

Table S.9. Ethylene chlorination and oxychlorination to EDC and cracking (BAU scenario) LCI.

**Functional unit: 1 kg VCM**		
**Input**	**Amount**	**Units**
Ethylene (market for ethylene)	$4.53·10^{-1}$	kg
Cl_2 (market for chlorine, gaseous)	$5.71·10^{-1}$	kg
O_2 (market for oxygen, liquid)	$1.35·10^{-1}$	kg
Water (market group for tap water)	$3.84·10^{-1}$	kg
Heating (market for heat, district or industrial, natural gas)	2.38	MJ
Cooling (water from 25 to 20 ºC)	4.11	MJ
Refrigeration (–50 ºC)	2.15	MJ
Electricity (market for electricity, high voltage)	$2.44·10^{-2}$	kWh
**Output**		
VCM	1.00	kg
Water (emission to water)	$4.41·10^{-3}$	m^3
CO_2 (emission to air)	$1.78·10^{-2}$	kg

Table S.10. Ethane chlorination to EDC and cracking (2-step real scenario) LCI.

Functional unit: 1 kg VCM		
**Input**	**Amount**	**Units**
Ethane (market for ethane)	$6.58 \cdot 10^{-1}$	kg
Cl_2 (market for chlorine, gaseous)	1.02	kg
O_2 (market for oxygen, liquid)	$4.04 \cdot 10^{-1}$	kg
Water (market group for tap water)	4.68	kg
Heating (market for heat, district or industrial, natural gas)	2.33	MJ
Cooling (water from 25 to 20 ºC)	$1.10 \cdot 10^{1}$	MJ
Refrigeration (–50 ºC)	$9.57 \cdot 10^{-1}$	MJ
Electricity (market for electricity, high voltage)	$4.32 \cdot 10^{-1}$	kWh
**Output**		
VCM	1.00	kg
EC	$3.54 \cdot 10^{-1}$	kg
Water (emission to water)	$1.04 \cdot 10^{-3}$	m^3
CO_2 (emission to air)	$5.98 \cdot 10^{-3}$	kg

Table S.11. Ideal ethane chlorination to EDC and cracking (2-step ideal scenario) LCI.

**Functional unit: 1 kg VCM**		
**Input**	**Amount**	**Units**
Ethane (market for ethane)	$4.93 \cdot 10^{-1}$	kg
Cl_2 (market for chlorine, gaseous)	$5.73 \cdot 10^{-1}$	kg
O_2 (market for oxygen, liquid)	$3.96 \cdot 10^{-1}$	kg
Water (market group for tap water)	4.20	kg
Heating (market for heat, district or industrial, natural gas)	2.23	MJ
Cooling (water from 25 to 20 ºC)	4.57	MJ
Refrigeration (–50 ºC)	1.87	MJ
Electricity (market for electricity, high voltage)	$3.29 \cdot 10^{-2}$	kWh
**Output**		
VCM	1.00	kg
Water (emission to water)	$6.39 \cdot 10^{-4}$	m^3
CO_2 (emission to air)	$3.85 \cdot 10^{-3}$	kg

Table S.12. Direct ethane chlorination to VCM (1-step real scenario) LCI.

**Functional unit: 1 kg VCM**		
**Input**	**Amount**	**Units**
Ethane (market for ethane)	$7.21 \cdot 10^{-1}$	kg
Cl_2 (market for chlorine, gaseous)	$7.88 \cdot 10^{-1}$	kg
O_2 (market for oxygen, liquid)	$5.06 \cdot 10^{-1}$	kg
Water (market group for tap water)	4.20	kg
Heating (market for heat, district or industrial, natural gas)	4.64	MJ
Cooling (water from 25 to 20 ºC)	4.57	MJ
Refrigeration (–50 ºC)	5.59	MJ
Refrigeration (–125 ºC)	1.50	MJ
Electricity (market for electricity, high voltage)	$3.71 \cdot 10^{-1}$	kWh
**Output**		
VCM	1.00	kg
Ethylene	$1.28 \cdot 10^{-1}$	kg
EC	$4.74 \cdot 10^{-2}$	kg
1,1-dichloroethane	$2.60 \cdot 10^{-1}$	kg
Water (emission to water)	$5.86 \cdot 10^{-3}$	m^3
CO_2 (emission to air)	$5.26 \cdot 10^{-3}$	kg

Table S.13. Ideal direct ethane chlorination to VCM (1-step ideal scenario) LCI.

Functional unit: 1 kg VCM		
**Input**	**Amount**	**Units**
Ethane (market for ethane)	$4.68 \cdot 10^{-1}$	kg
Cl_2 (market for chlorine, gaseous)	$5.92 \cdot 10^{-1}$	kg
O_2 (market for oxygen, liquid)	$3.73 \cdot 10^{-1}$	kg
Water (market group for tap water)	3.95	kg
Heating (market for heat, district or industrial, natural gas)	$7.38 \cdot 10^{-1}$	MJ
Cooling (water from 25 to 20 ºC) (ref)	$4.55 \cdot 10^{-1}$	MJ
Refrigeration (–50 ºC)	$9.55 \cdot 10^{-1}$	MJ
Refrigeration (–125 ºC)	-	MJ
Electricity (market for electricity, high voltage)	$1.75 \cdot 10^{-1}$	kWh
**Output**		
VCM	1.00	kg
Water (emission to water)	$4.20 \cdot 10^{-4}$	m^3
CO_2 (emission to air)	-	kg

Table S.14. HCl oxidation to Cl_2 (Deacon process) LCI.

**Functional unit: 1 kg Cl_2**		
**Input**	**Amount**	**Units**
HCl 30 wt.% (dry base)	1.03	kg
O_2 (market for oxygen, liquid)	$2.26 \cdot 10^{-1}$	kg
Heating (market for heat, district or industrial, natural gas)	$3.86 \cdot 10^{-1}$	MJ
Cooling (water from 25 to 20 ºC)	$8.24 \cdot 10^{-1}$	MJ
**Output**		
Cl_2	1.00	kg
Water (emission to water)	$2.54 \cdot 10^{-4}$	m^3

Table S.15. Cooling water (25 to 20 ºC) LCI.[10]

Functional unit: 1 MJ cooling		
**Input**	**Amount**	**Units**
Water (market group for tap water)	$6.11 \cdot 10^{-1}$	kg
Electricity (market for electricity, high voltage)	$1.66 \cdot 10^{-3}$	kWh
**Output**		
Water (emission to air)	$4.08 \cdot 10^{-4}$	m^3
Water (emission to water)	$2.04 \cdot 10^{-4}$	m^3

Table S.16. Refrigeration cascade cycle NH_3-CO_2 at –50 ºC LCI.

Functional unit: 1 MJ cooling		
**Input**	**Amount**	**Units**
Cooling (water from 25 to 20 ºC)	1.83	MJ
Electricity (market for electricity, high voltage)	$2.30 \cdot 10^{-1}$	kWh

Table S.17. Refrigeration cascade cycle propene-ethylene-methane at –125 ºC LCI.[11,12]

Functional unit: 1 MJ cooling		
**Input**	**Amount**	**Units**
Cooling (water from 25 to 20 ºC)	3.99	MJ
Electricity (market for electricity, high voltage)	$8.29 \cdot 10^{-1}$	kWh

C.2. Damage assessment

In this section, we present the endpoints of the ReCiPe 2016 v1.1 method as supplementary LCA information (**Figures S.8-S.10**).

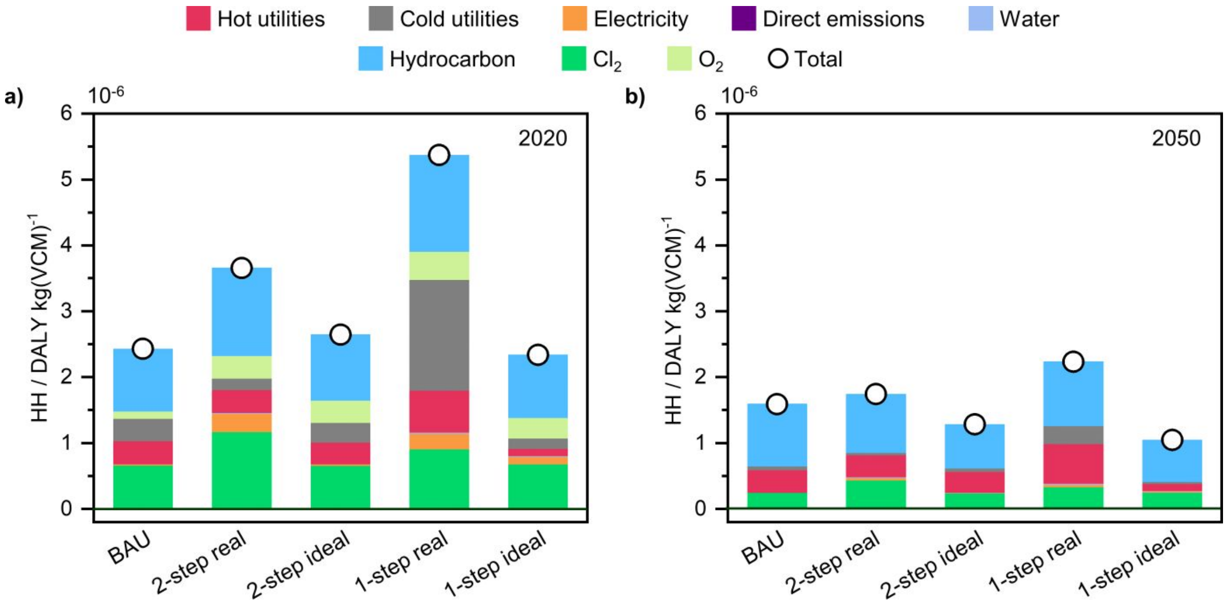

Figure S.6. Human health (HH) endpoint results for VCM synthesis. **a)** Present 2020 scenario. **b)** Future 2050 scenario.

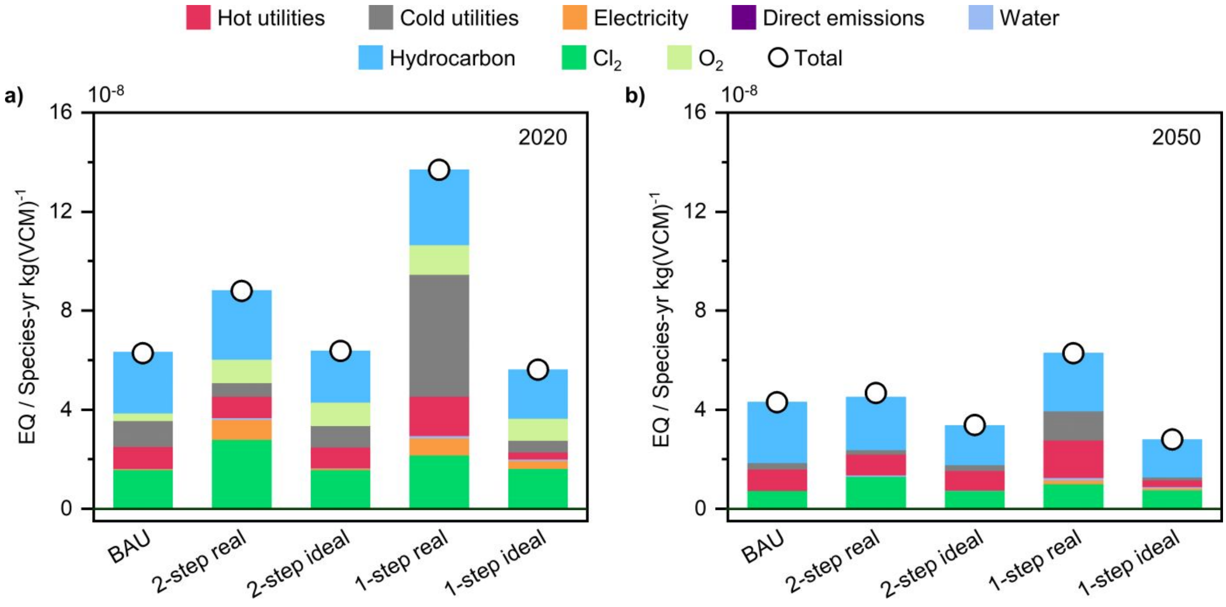

Figure S.7. Ecosystems quality (EQ) endpoint results for VCM synthesis. **a)** Present 2020 scenario. **b)** Future 2050 scenario.

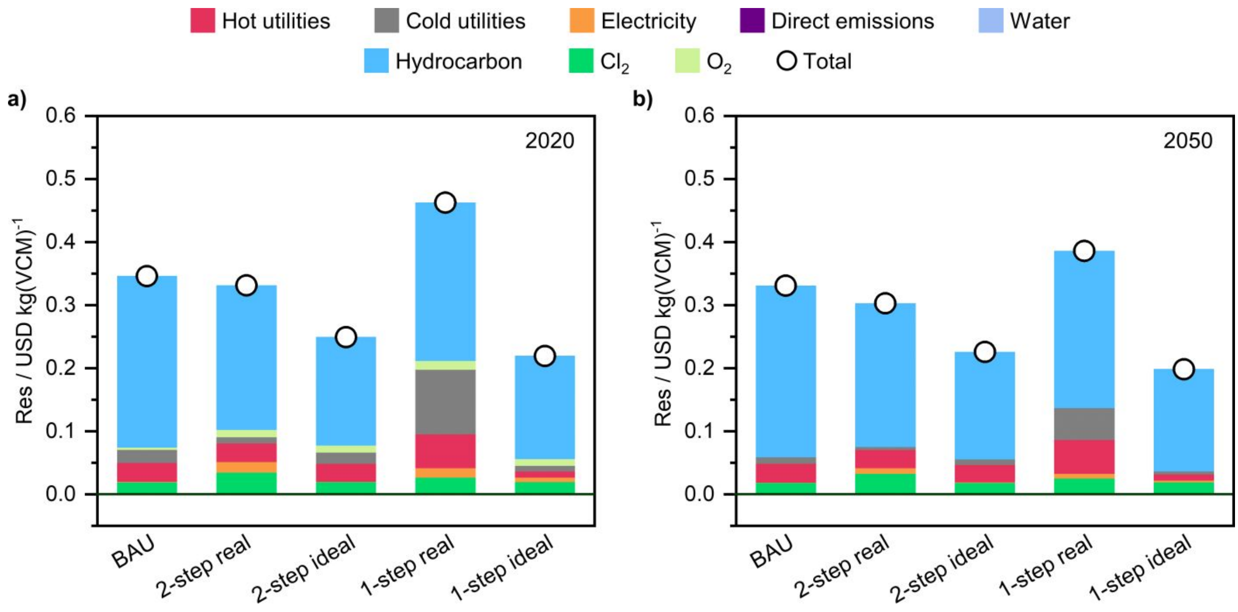

Figure S.8. Resource scarcity (RS) endpoint results for VCM synthesis. **a)** Present 2020 scenario. **b)** Future 2050 scenario.

D. Economic assessment

In this section, we provide the data employed in the economic study.

D.1. Variable operating costs

Table S.18. Material and utility prices.

Input	Amount	Units	Reference
Ethylene	1.26	$\$\cdot kg^{-1}$	13
Ethylene (2050)*	2.92	$\$\cdot kg^{-1}$	14
Ethane	$2.00\cdot10^{-1}$	$\$\cdot kg^{-1}$	13
Cl_2	$2.84\cdot10^{-1}$	$\$\cdot kg^{-1}$	15
O_2	$1.23\cdot10^{-1}$	$\$\cdot kg^{-1}$	16
Water	$1.77\cdot10^{-4}$	$\$\cdot kg^{-1}$	17
Heating (natural gas)	$3.51\cdot10^{-3}$	$\$\cdot MJ^{-1}$	17
Cooling (water from 25 to 20 ºC)	$3.78\cdot10^{-4}$	$\$\cdot MJ^{-1}$	17
Refrigeration (–50 ºC)	$2.60\cdot10^{-2}$	$\$\cdot MJ^{-1}$	**
Refrigeration (–125 ºC)	$9.27\cdot10^{-2}$	$\$\cdot MJ^{-1}$	**
Electricity	$1.10\cdot10^{-1}$	$\$\cdot kWh^{-1}$	18

*Calculated by following the trend in the Annual Energy Outlook 2021.[14]

**Calculated from the respective inventory found in Section C.1.

D.2. Capital investment costs

The fixed capital cost (total fixed capital cost, *CAPEX*) is based on Sinnot and Towler.[9] First, we estimate the purchased equipment costs from the capacity using Eq.(21):

$$C_{e,i} = a_i + b_i S_i^{n_i} \tag{21}$$

Where $C_{e,i}$ is the purchased equipment cost of i in 2010 US \$, a_i, b_i and n_i are constants, and S_i is the size parameter measured in different units depending on the type of equipment i. These data can be found in Sinnot and Towler.[9]

Then, we update these costs from 2010 to 2019 using the Chemical Engineering Plant Cost Index (CEPCI) (Eq.(22)):

$$\text{Cost in year A} = \text{Cost in year B}\frac{\text{Cost index in year A}}{\text{Cost index in year B}} \tag{22}$$

Where Cost in year B and Cost in year A are the purchased equipment costs [\$] for the years 2010 and 2019 and $\text{Cost index in year B}$ (532.9) and $\text{Cost index in year A}$ (607.5) are the CEPCI annual mean values for the corresponding years.

With the updated purchased equipment cost, we add the factors for equipment erection (f_{er} = 0.3), piping (f_p = 0.8) instrumentation and process control (f_i = 0.3), electrical work (f_{el} = 0.2), civil engineering work (f_c = 0.3), structures and buildings (f_s = 0.2), lagging, insulation or paint (f_l = 0.1) and material factor (f_m = 1.0 for carbon steel) to calculate the installed capital cost (C, \$) using Eq.(23):

$$C = \sum_i C_{e,i}\left[(1+f_p) + \frac{f_{er}+f_{el}+f_i+f_c+f_s+f_l}{f_m}\right] \tag{23}$$

Then, we add the offsites (*OS* = 0.3), design and engineering (*D&E* = 0.3), and contingency (*X* = 0.1) to get the *CAPEX* [\$] (Eq.(24)):

$$CAPEX = C(1+OS)(1+D\&E+X) \tag{24}$$

We calculate the annualization factor (or annual capital charge ratio, *ACCR*) considering a 30-year lifetime of the plant and an interest rate of 0.1 as described in Eq.(25):

$$ACCR = \frac{\left[i(1+i)^n\right]}{\left[(i+1)^n - 1\right]} \tag{25}$$

Finally, the product of the *ACCR* and the *CAPEX* results in the annualized capital investment (*ACC*, \$/y) (Eq.(26)):

$$ACC = CAPEX \cdot ACCR \tag{26}$$

In order to address the potential benefits of economy of scale, we apply the cost curve method (Eq.(27)) to scale the *CAPEX* based on the production rate of the plant:

$$C_2 = C_1 \left(\frac{S_2}{S_1} \right)^n \qquad (27)$$

Where C_1 and C_2 are the *CAPEX* [$/y] values of the plant with capacity [kt/y] S_1 and S_2, respectively; and n is a parameter that typically averages 0.6 across the whole chemical industry.

In **Table S.19**, we present the results for plants operating at 300 and 1900 kt/y, which is the range found in the VCM industry in the US[3]:

Table S.19. *CAPEX, ACC/*capacity and total production cost per kg of VCM.

**Capacity**	**300 kt/y**			**1900 kt/y**		
**Technology**	***CAPEX* [M$]**	***ACC*/capacity [$/kg]**	**Total cost [$/kg]**	***CAPEX* [M$]**	***ACC*/capacity [$/kg]**	**Total cost [$/kg]**
BAU	7.93	0.03	0.85	24.0	0.01	0.83
2-step real	28.7	0.10	0.65	87.0	0.04	0.59
2-step ideal	8.40	0.03	0.40	25.4	0.01	0.38
1-step real	18.8	0.06	0.83	56.9	0.03	0.80
1-step ideal	8.49	0.03	0.39	25.7	0.01	0.37

E. References

(1) Zichittella, G.; Pérez-Ramírez, J. Ethane-Based Catalytic Process for Vinyl Chloride Manufacture. *Angew. Chemie* **2021**, *133* (45), 24291. https://doi.org/10.1002/ange.202105851.

(2) Guedes, B. P.; Feitosa, M. F.; Vasconcelos, L. S.; Araújo, A. B.; Brito, R. P. Sensitivity and Dynamic Behavior Analysis of an Industrial Azeotropic Distillation Column. *Sep. Purif. Technol.* **2007**, *56* (3), 270. https://doi.org/10.1016/j.seppur.2007.02.014.

(3) Dimian, A. C.; Bildea, C. S. *Chemical Process Design: Computer Aided Case Studies*; John Wiley & Sons, 2008. https://doi.org/10.1002/9783527621583.

(4) Dreher, E.-L.; Beutel, K. K.; Myers, J. D.; Lübbe, T.; Krieger, S.; Pottenger, L. H. Chloroethanes and Chloroethylenes. *Ullmann's Encycl. Ind. Chem.* **2014**, 1. https://doi.org/10.1002/14356007.o06_o01.pub2.

(5) Lakshmanan, A.; Rooney, W. C.; Biegler, L. T. A Case Study for Reactor Network Synthesis: The Vinyl Chloride Process. *Comput. Chem. Eng.* **1999**, *23* (4-5), 479. https://doi.org/10.1016/S0098-1354(98)00287-7.

(6) Papp, R. Organochlorine Waste Management. *Pure Appl. Chem.* **1996**, *68* (9), 1801. https://doi.org/10.1351/pac199668091801.

(7) Hibi, T.; Nishida, H.; Hiroaki, A. Process for Producing Chlorine (US5871707A), 1999.

(8) Seki, K. Development of RuO_2/Rutile-TiO_2 Catalyst for Industrial HCl Oxidation Process. *Catal. Surv. from Asia* **2010**, *14* (3-4), 168. https://doi.org/10.1007/s10563-010-9091-7.

(9) Huijbregts, M. A. .; Steinmann, Z. J. .; Elshout P.M.F; Stam, G.; Verones, F.; Vieira, M. D. .; Hollander, A.; Zijp, M.; van Zelm, R. ReCiPe 2016 v1.1. *RIVM Rep. 2016-0104* **2017**, 201.

(10) Charalambous, M. A.; Tulus, V.; Ryberg, M. W.; Pérez-Ramírez, J.; Guillén-Gosálbez, G. Absolute Environmental Sustainability Assessment of Renewable Dimethyl Ether Fuelled Heavy-Duty Trucks. *Sustain. Energy Fuels* **2023**, 1930. https://doi.org/10.1039/d2se01409b.

(11) Ioannou, I.; Javaloyes-Antón, J.; Caballero, J. A.; Guillén-Gosálbez, G. Economic and Environmental Performance of an Integrated CO_2 Refinery. *ACS Sustain. Chem. Eng.* **2023**, *11* (5), 1949. https://doi.org/10.1021/acssuschemeng.2c06724.

(12) Luyben, W. L. Estimating Refrigeration Costs at Cryogenic Temperatures. *Comput. Chem. Eng.* **2017**, *103*, 144. https://doi.org/10.1016/j.compchemeng.2017.03.013.

(13) Dutta, A.; Karimi, I. A.; Farooq, S. Technoeconomic Perspective on Natural Gas Liquids

and Methanol as Potential Feedstocks for Producing Olefins. *Ind. Eng. Chem. Res.* **2019**, *58* (2), 963. https://doi.org/10.1021/acs.iecr.8b05277.

(14) U.S. Energy Information Administration. *Appendix A. Total energy supply, disposition, and price summary*. Annual Energy Outlook 2021. https://www.eia.gov/outlooks/aeo/pdf/appa.pdf.

(15) Richard A., T.; Shaeiwitz, J. A.; Bhattacharyya, D.; Wallace B., W. 8.4 Raw Material Costs. In *Analysis, Synthesis and Design of Chemical Processes*; Prentice Hall, 2018.

(16) Cong, L.; Chang, L.; Liu, X.; Deng, X.; Chen, H. Analysis of CO_2 Emission and Economic Feasibility for a Heat-Integrated Air Separation System. *Chem. Eng. Technol.* **2018**, *41* (8), 1639. https://doi.org/10.1002/ceat.201600052.

(17) Richard A., T.; Shaeiwitz, J. A.; Bhattacharyya, D.; Wallace B., W. 8.3 Utility Costs. In *Analysis, Synthesis and Design of Chemical Processes*; Prentice Hall, 2018.

(18) U.S. Energy Information Administration. *Electricity Production Costs Rose with Fuel Costs in 2021, Delivery Cost Remained Elevated*. Today in Energy. https://www.eia.gov/todayinenergy/detail.php?id=55639.

(19) Towler, G.; Sinnott, R. K. Chemical Engineering Design; Elsevier Ltd, 2013. https://doi.org/10.1016/C2009-0-61216-2.
